# Bat-derived antimicrobial peptide MC-CATH3 Confers protection against bacterial infections *via* a potential ferroptosis-like death process

**DOI:** 10.1016/j.jbc.2026.113456

**Published:** 2026-08-17

**Authors:** Xiangjin Kong, Zhouye Xu, Jinhui Li, Zhizhen Liu, Yiyun Zhu, Zifan Ye, Shuangyu Li, Jingjing Zhang, Lei Fu, Xudong Jiao, Yipeng Wang

**Affiliations:** 1 Yantai Institute of Coastal Zone Research, Chinese Academy of Sciences, Yantai, Shandong, China; 2 University of Chinese Academy of Sciences, Beijing, China; 3 Department of Biopharmaceutical Sciences, College of Pharmaceutical Sciences, Soochow University, Suzhou, Jiangsu, China; 4 Institute of Medical Artificial Intelligence, South China Hospital, Medical School, Shenzhen University, Shenzhen, Guangdong, China; 5 School of Biological Science and Technology, University of Jinan, Jinan, Shandong, China

**Keywords:** antimicrobial peptide, reactive oxygen species, antimicrobial mechanism, ferroptosis-like death, cathelicidin

## Abstract

Most naturally occurring antimicrobial peptides (AMPs) exert bactericidal activity by disrupting bacterial membranes, and although alternative mechanisms have been described, AMPs associated with ferroptosis-like features in bacterial killing remain rare. Here, we identified three novel AMPs, MC-CATH1-3, from the bat *Myotis chinensis.* All three peptides adopt an α-helical conformation and display potent antimicrobial activity. Notably, compared with MC-CATH1/2, MC-CATH3 causes weak membrane perturbation but triggers metabolic imbalance and excessive production of reactive oxygen species, accompanied by iron–sulfur cluster damage and abnormal intracellular Fe^2+^ accumulation. These events are associated with Fenton reaction–driven lipid peroxidation and are consistent with a potential ferroptosis-like bacterial death process. *In vivo*, MC-CATHs, especially MC-CATH3, exhibit remarkable therapeutic efficacy in cutaneous wound infection. Collectively, our findings broaden the conceptual framework of AMP mechanisms and suggest that MC-CATH3 may serve as a promising template for the rational design of peptide antibiotics with novel modes of action.

The relentless rise of antibiotic-resistant bacteria has become one of the most pressing global health crises. In 2019 alone, antimicrobial resistance (AMR) claimed millions of lives worldwide, exceeding the combined mortality of AIDS and malaria (1). Recognizing the urgency of this threat, the World Health Organization (WHO) revised its Bacterial Priority Pathogens List (BPPL) in 2024, designating 15 antibiotic-resistant pathogens and ranking them into critical, high, and medium priority groups. Yet, the therapeutic arsenal remains dangerously limited: carbapenems and fluoroquinolones, once regarded as last-resort agents, are increasingly ineffective against resistant strains (2, 3, 4). Antibiotic discovery, however, has largely stagnated since the 1990s, underscoring the critical need for alternative antimicrobial strategies.

Antimicrobial peptides (AMPs) have emerged as promising therapeutic candidates in this context. Ubiquitously distributed across nearly all species of life, naturally occurring AMPs—particularly those adopting α-helical conformations—exhibit potent antimicrobial activity, primarily attributed to their capacity to disrupt bacterial membranes (5). Structural transitions upon contact with anionic membrane components enhance their affinity for lipid bilayers, leading to permeabilization and leakage of cellular contents (6, 7, 8). This process is influenced by conformational changes in the AMPs and the peptide-lipid ratio (9, 10, 11). Upon encountering anionic lipid components, AMPs transition from a disordered conformation in aqueous environments to an amphipathic α-helical structure, thereby strengthening membrane interactions and antimicrobial potency. Consequently, α-helical AMPs generally display potent antimicrobial activity. In contrast, β-sheet AMPs, stabilized by intramolecular disulfide bonds, exhibit limited conformational flexibility and undergo minimal structural changes upon membrane contact. In addition, the peptide-to-lipid ratio is a critical determinant of AMP behavior, with higher ratios facilitating deeper insertion into hydrophobic membrane domains and enhancing membrane disruption. (9). These interactions have been conceptualized into several models of action, including barrel-stave, toroidal pore, carpet, and aggregate models (12).

In addition to membrane disruption, an increasing body of evidence has revealed the intracellular targeting capabilities of AMPs. Buforin II, a 21-residue peptide derived from buforin I of the Asian toad *Bufo garagrizans*, exhibits potent antimicrobial activity by penetrating bacterial membranes and binding nucleic acids, thereby killing bacteria without causing membrane lysis (13). Similarly, indolicidin, an AMP enriched in tryptophan and proline from bovine neutrophil granules, interacts with genomic DNA and inhibits replication (14). The human neutrophil peptide HNP-1 targets lipid precursors in the cell wall of *Staphylococcus aureus*, effectively blocking cell wall synthesis (15). Proline-rich AMPs (PrAMPs) of the cathelicidin family, including Bac5 and Bac7 from even-toed ungulates, bypass membrane disruption and instead suppress protein synthesis. Despite sequence diversity, these PrAMPs bind overlapping regions of the ribosomal exit tunnel and inhibit translation by either blocking the transition from initiation to elongation or preventing release factor dissociation during termination (16, 17). The human cathelicidin LL-37, widely known for its immunomodulatory and membrane-disruptive properties, also exhibits intracellular targeting activity by inhibiting the palmitoyltransferase PagP in *Escherichia coli* (18). Recent studies have further highlighted the role of reactive oxygen species (ROS) in antimicrobial action. Under stress from antimicrobial agents, bacteria often accumulate high levels of ROS, which can persist even after the removal of the stressor and contribute to bacterial eradication (19). Consistently, several AMPs have been shown to promote ROS generation. For example, the hybrid peptide CM15 elevates superoxide anion, hydroxyl radical, and hydrogen peroxide within *E. coli* cells (20), while LL-37 induces bursts of ROS that disrupt metabolic processes associated with active respiration (21).

Ferroptosis is a unique form of programmed cell death driven by lipid peroxidation and closely associated with iron metabolism and redox imbalance. This process is orchestrated by diverse metabolic pathways involving amino acids, lipids, and sugars, and is tightly coupled to redox homeostasis, iron accumulation, and mitochondrial respiratory activity (22). Dysregulated ferroptosis has been implicated in the development of multiple organ injuries and degenerative diseases (23, 24). In microbial systems, ferroptosis-like death has been proposed to involve ROS accumulation, lipid peroxidation, and impairment of glutathione peroxidase-associated antioxidant defenses (25).

Although thousands of naturally occurring AMPs have been identified from diverse organism species, natural AMPs capable of engaging bacterial killing with ferroptosis-like features remain rare. The only reported example is Urechistachykinin I, a natural AMP identified from the marine worm *Urechis unicinctus*. It has recently been shown to induce mitochondrial and metabolic dysfunctions that culminate in ferroptosis-like, putatively iron-associated cell death in the microorganisms *Saccharomyces cerevisiae* and *Vibrio vulnificus* (26, 27). Although previous studies have suggested a link between natural antimicrobial peptides and oxidative phenotypes associated with iron dysregulation in bacteria, their conclusions were primarily supported by hallmark-based observations rather than a fully delineated mechanistic framework. In particular, a causal sequence connecting the initial peptide–bacterium interaction to iron dysregulation, lipid peroxidation, and death execution has not been comprehensively established. As a result, it remains uncertain whether iron-associated lipid peroxidation functions as a primary driver of bacterial death or instead represents a downstream consequence of broader oxidative stress responses. Given that reactive oxygen species accumulation and membrane perturbation are common outcomes of multiple antimicrobial mechanisms, the molecular and metabolic criteria required to define a structured ferroptosis-like or iron-associated oxidative death process in bacteria remain to be systematically clarified. Here, we report the identification of three novel cathelicidin-family AMPs (MC-CATH1-3) from the bat *Myotis chinensis*. These peptides exhibit broad-spectrum antimicrobial and antibiofilm activities with favorable cytocompatibility, yet their mechanisms of action are markedly distinct. Notably, MC-CATH3 shows minimal capacity for membrane disruption and DNA binding but strongly induces ROS accumulation. Mechanistic analyses suggest that MC-CATH3 triggers a cascade of oxidative events associated with iron dysregulation, including disruption of iron-sulfur clusters, intracellular Fe^2+^ accumulation, and Fenton reaction-related lipid peroxidation, consistent with a potential ferroptosis-like death process in bacteria. In a mouse skin wound infection model, further evaluation of their therapeutic potential revealed that all MC-CATHs effectively controlled infection and promoted wound healing, with MC-CATH3 exhibiting the most pronounced efficacy and achieving overall therapeutic outcomes comparable to vancomycin. This study suggests that ferroptosis-like features may contribute to the antibacterial activity of certain AMPs and provides a conceptual basis for exploring redox-targeted peptide therapeutics against multidrug-resistant pathogens.

## Results

### Identification and characterization of MC-CATHs

A lung cDNA library of *M. chinensis* was successfully constructed, and three novel cathelicidin family AMP cDNA sequences (GenBank accession numbers: PQ661215-PQ661217) were identified. The complete nucleotide and deduced amino acid sequences of the precursors are shown in Fig. S1. The cDNA sequences encoding the MC-CATH1-3 precursors were 606, 606, and 603 bp in length, respectively. The translated peptide precursors comprised 168, 172, and 180 amino acid residues, respectively. The putative mature cleavage sites of these peptides were predicted based on conserved processing motifs reported in other members of the cathelicidin family. MC-CATH1 consists of 36 amino acid residues, including nine basic residues (1 Lys, 8 Arg) and three acidic residues (2 Asp, 1 Glu), with the sequence GLKARAGRLPGLFGLLWDRIRNRGRRPRDVFENLSA. MC-CATH2 comprises 40 amino acid residues, including 10 basic residues (1 Lys, 9 Arg) and four acidic residues (1 Asp, 3 Glu), with the sequence GLKARVLRVNNVFRRLWDTLRNRGWRPRLFIRNLSPEEEP. MC-CATH3 contains 42 amino acid residues, including 13 basic residues (9 Lys, 4 Arg) and six acidic residues (6 Glu), with the sequence KLNAENLGERIKNAKKKVWEKIKSFGRRIKEFFRKPSPEGEP. BLAST analysis against the NCBI protein database showed that MC-CATH1 and MC-CATH3 share significant sequence homology with cathelicidin-related precursor proteins/proproteins reported in other bat species, including *Myotis daubentonii*, *Myotis davidii*, *Myotis brandtii*, *Myotis yumanensis*, and *Myotis nattereri*. Notably, most of the homologous sequences were annotated as predicted proteins or precursor proteins rather than experimentally validated mature antimicrobial peptides. These findings suggest that MC-CATH1 and MC-CATH3 are bat cathelicidin-related peptides that remain poorly characterized at the functional level.

### MC-CATHs are mainly composed of α-helical structures

The physical and chemical parameters of MC-CATH1-3 were analyzed using the ExPASy Bioinformatics Resource Portal (http://www.expasy.org/tools/) (Table 1). All three peptides exhibited a high abundance of basic residues (9, 10, and 13, respectively). Their theoretical isoelectric points (pI > 10) indicated strong cationic character under physiological conditions, with net charges of +6, +6, and +7, respectively.

**Table 1:** Physicochemical parameters of MC-CATH1-3

Peptide	Number of amino acids (n)	Molecular weight	Net charge	Theoretical pI	Grand average of hydropathicity
MC-CATH1	36	4134.8	+6	12.4	−0.61
MC-CATH2	40	4931.7	+6	12.3	−0.85
MC-CATH3	42	4984.8	+7	10.8	−1.33

**Figure 1:**
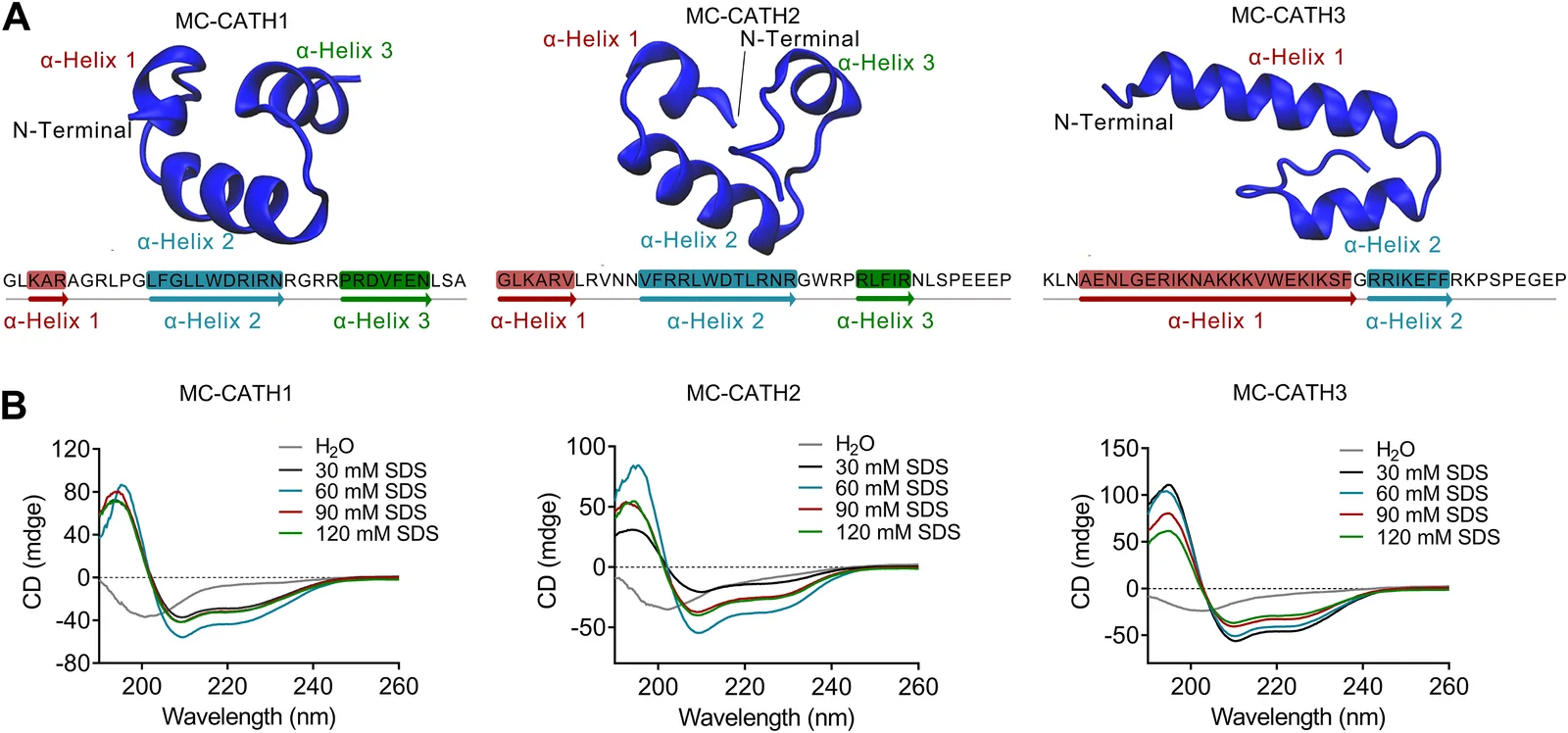
**Secondary structure analysis of the MC-CATHs.***A*, Predicted structures of MC-CATHs generated by PEP-FOLD3.5 (https://mobyle.rpbs.univ-paris-diderot.fr/cgi-bin/portal.py#forms::PEP-FOLD3). Amino acid sequences, peptide N-termini, and helix numbers are indicated. *B*, Circular dichroism (CD) spectra of MC-CATHs in different solvent environments. Peptides were dissolved in 0, 30, 60, 90, and 120 mM SDS micelles at a concentration of 0.2 mg/ml. Experiments were repeated three times independently, and one representative spectrum is shown.

Secondary structure prediction by PEP-FOLD3.5 suggested that MC-CATH1-3 predominantly adopt α-helical conformations (Fig. 1*A*). SOPMA analysis further estimated α-helix contents of 55.56%, 47.50%, and 78.57% for MC-CATH1, 2, and 3, respectively. To validate these predictions, circular dichroism (CD) spectroscopy was performed. In aqueous solution, the CD spectra of all three peptides exhibited a prominent negative peak at 200 nm, consistent with a random-coil conformation (Fig. 1*B*). In contrast, exposure to membrane-mimetic SDS micelles (30–120 mM) induced characteristic α-helical spectral signatures, with an intense positive peak at 190 nm and two negative minima at 208 and 222 nm. Together, these data indicate that MC-CATH1-3 undergo structural transitions toward α-helicity in membrane-like environments, reflecting their conformational plasticity and amphipathic nature.

### MC-CATHs exhibited broad-spectrum, high-efficiency antibacterial and anti-biofilm activities

The antimicrobial potency of MC-CATH1-3 was determined using a 2-fold broth microdilution method. All three peptides displayed potent, broad-spectrum activity against 13 clinically relevant pathogens (6 Gram-positive bacteria, 5 Gram-negative bacteria, and two fungi) (Table 2). MC-CATH1 exhibited minimum inhibitory concentrations (MICs) ranging from 0.19 to 18.14 μM for bacteria and 9.07 to 18.14 μM for fungi. MC-CATH2 showed MICs ranging from 0.16 to 15.21 μM for bacteria and 3.8 μM for *C. albicans* 08090102. MC-CATH3 demonstrated MICs ranging from 0.47 to 7.52 μM for most of the tested bacteria and 3.76 μM for *C. albicans* 08090102, but exhibited no detectable activity against *B. cere*us CMCC63301, *Saccharomyces epidermidis* CI, *E. coli* CMCC44102, and *C. glabrata* CI. Comparison of MICs and geometric mean (GM) values revealed that MC-CATH2 exhibited the highest overall potency, while MC-CATH1 showed the widest antimicrobial spectrum among the three peptides but weaker activity than MC-CATH3.

**Table 2:** Antimicrobial activities of MC-CATH1-3

Microorganisms	MIC (μg/ml)						
	MC-CATH1	MC-CATH2	MC-CATH3	Ampicillin	Polymyxin B	Vancomycin	Meropenem
Gram-positive bacteria							
*E. faecalis* ATCC29217	0.78 (0.19 μM)	0.78 (0.16 μM)	2.34 (0.47 μM)	0.04 (0.1 μM)	9.38 (7.21 μM)	>100 (>67.3 μM)	0.04 (0.1 μM)
*B. subtilis* CMCC63501	18.75 (4.53 μM)	9.38 (1.90 μM)	9.38 (1.88 μM)	9.38 (26.85 μM)	75 (57.62 μM)	75 (50.48 μM)	0.15 (0.39 μM)
*B. cereus* CMCC63301	75 (18.14 μM)	75 (15.21 μM)	>100 (>20.06 μM)	>100 (>286.2 μM)	75 (57.62 μM)	75 (50.48 μM)	0.01 (0.03 μM)
*S. aureus* CMCC26003	9.38 (2.27 μM)	9.38 (1.90 μM)	4.69 (0.94 μM)	0.04 (0.1 μM)	37.5 (28.81 μM)	0.59 (0.4 μM)	0.04 (0.1 μM)
*C. perfringen* ATCC13124	18.75 (4.53 μM)	18.75 (3.80 μM)	37.5 (7.52 μM)	2.34 (6.7 μM)	9.38 (7.21 μM)	37.5 (25.24 μM)	0.02 (0.05 μM)
*S. epidermidis* CI	75 (18.14 μM)	18.75 (3.80 μM)	>100 (>20.06 μM)	2.34 (6.7 μM)	>100 (>76.83 μM)	>100 (>67.3 μM)	0.07 (0.19 μM)
Gram-negative bacteria							
*A. baumannii* ATCC19606	4.69 (1.13 μM)	4.69 (0.95 μM)	4.69 (0.94 μM)	>100 (>286.2 μM)	1.17 (0.9 μM)	>100 (>67.3 μM)	0.15 (0.39 μM)
*E. coli* ATCC25922	9.38 (2.27 μM)	9.38 (1.90 μM)	9.38 (1.88 μM)	9.38 (26.85 μM)	4.69 (3.6 μM)	>100 (>67.3 μM)	0.02 (0.05 μM)
*E. coli* CMCC44102	75 (18.14 μM)	37.5 (7.60 μM)	>100 (>20.06 μM)	2.34 (6.7 μM)	2.34 (1.8 μM)	>100 (>67.3 μM)	1.17 (3.05 μM)
*P. aeruginosa* CMCC10104	18.75 (4.53 μM)	9.38 (1.90 μM)	9.38 (1.88 μM)	>100 (>286.2 μM)	2.34 (1.8 μM)	>100 (>67.3 μM)	1.17 (3.05 μM)
*S. castellani* ATCC12022	37.5 (9.07 μM)	4.69 (0.95 μM)	9.38 (1.88 μM)	1.17 (3.35 μM)	75 (57.62 μM)	75 (50.48 μM)	0.29 (0.76 μM)
Fungi							
*C. glabrata CI*	75 (18.14 μM)	>100 (>20.28 μM)	>100 (>20.06 μM)	>100 (>286.2 μM)	4.69 (3.6 μM)	>100 (>67.3 μM)	0.29 (0.76 μM)
*C. albican*s 08090102	37.5 (9.07 μM)	18.75 (3.80 μM)	18.75 (3.76 μM)	>100 (>286.2 μM)	18.75 (14.41 μM)	>100 (>67.3 μM)	0.01 (0.03 μM)
GM	20.22 (4.89 μM)	12.79 (2.7 μM)	18.42 (3.69 μM)	6.22 (17.45 μM)	13.20 (10.14 μM)	58.47 (39.37 μM)	0.08 (0.22 μM)

MIC values exceeding 100 μg/ml were defaulted to 100 μg/ml in the GM value calculation. MIC values were determined by a twofold broth microdilution assay in three independent experiments.

GM, Geometic mean of the MICs; CI, Clinical isolates.

**Figure 2:**
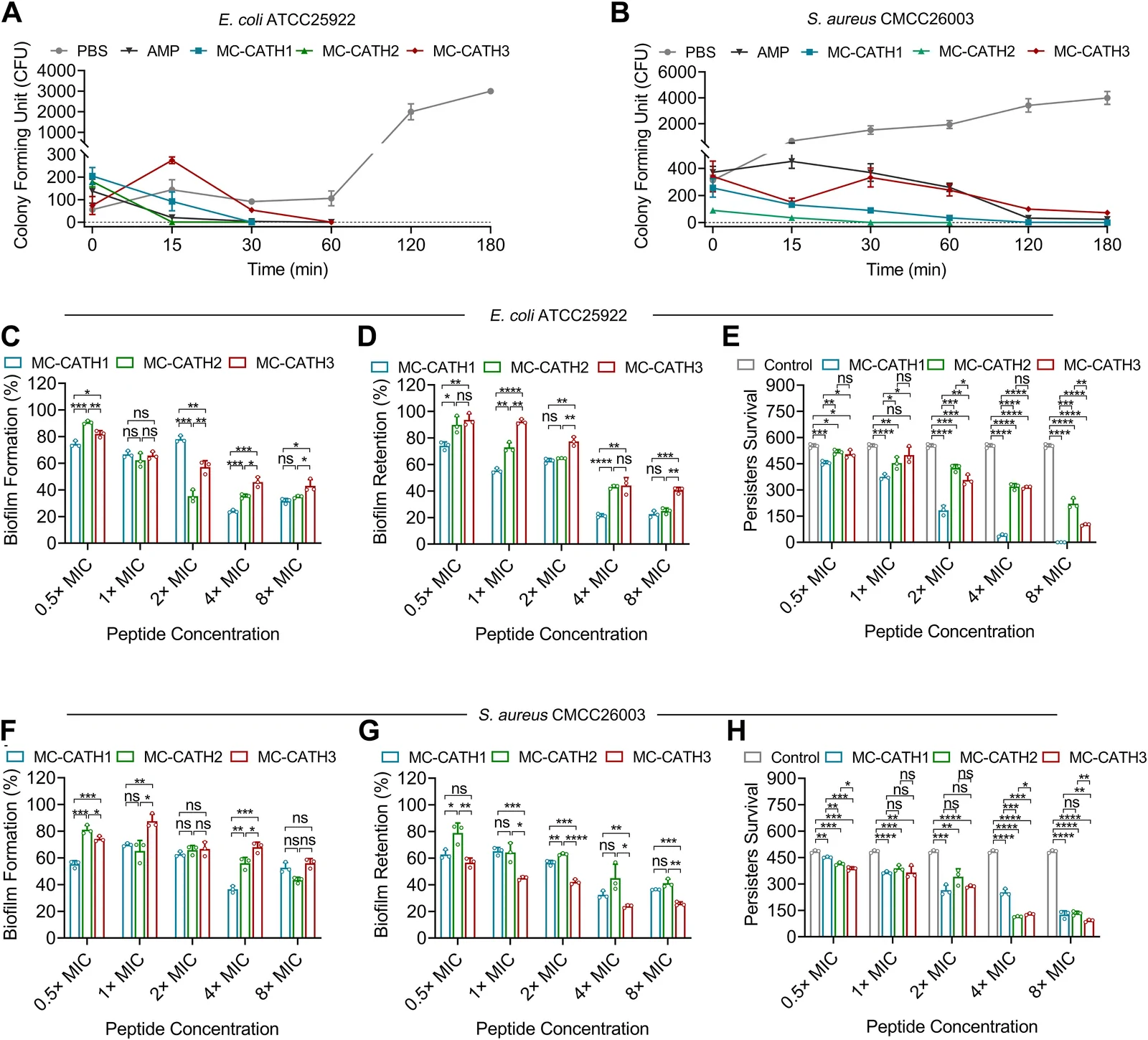
**Killing kinetics, anti-biofilm, and anti-persister activities of MC-CATHs.***A–B*, Time-kill kinetics of MC-CATHs against *E. coli* ATCC25922 (*A*) and *S. aureus* CMCC26003 (*B*). Bacteria were treated with peptides at 5 × MIC, and samples were collected at 0, 15, 30, 60, 120, and 180 min for CFU/ml quantification. AMP: ampicillin. *C*–*E*, Effects of MC-CATHs on *E. coli* ATCC25922: inhibition of biofilm formation (*C*), eradication of preformed biofilms (*D*), and elimination of persister cells (*E*). *F*–*H*, Effects of MC-CATHs on *S. aureus* CMCC26003: inhibition of biofilm formation (*F*), eradication of preformed biofilms (*G*), and elimination of persister cells (*H*). All experiments were performed in triplicate, and data are presented as mean ± SD. Statistical significance was assessed by unpaired *t* test (ns: not significant; ∗*p* < 0.05; ∗∗*p* < 0.01; ∗∗∗*p* < 0.001; ∗∗∗∗*p* < 0.0001).

To further evaluate bactericidal activity, time-kill assays were performed against *E. coli* ATCC25922 and *S. aureus* CMCC26003. At 5 × MIC, MC-CATH1-3 completely eradicated *E. coli* ATCC25922 within 60 min (Fig. 2*A*). Against *S. aureus*, MC-CATH2 achieved complete killing within 60 min, MC-CATH1 required 180 min to achieve full eradication, and MC-CATH3 eliminated 98% of the bacterial population within 180 min (Fig. 2*B*). Both MC-CATH1 and MC-CATH2 exhibited faster killing kinetics than the positive control ampicillin, whereas MC-CATH3 displayed the slowest bactericidal activity and showed comparable efficacy to ampicillin. The co-expression of MC-CATH1-3 in bat lung tissue prompted us to test whether these peptides display functional cooperation. To investigate this further, checkerboard assays were performed to assess synergistic interactions among the MC-CATH peptides. The results confirmed significant antibacterial synergy between MC-CATH1-3 peptides, with fractional inhibitory concentration (FIC) indices ≤0.5 (Fig. S2, *A*–*F*).

Because biofilms can protect bacteria from antimicrobial exposure and contribute to persistent infection (28, 29), we next investigated the anti-biofilm and anti-persister activities of MC-CATH1-3. The results demonstrate that MC-CATH1-3 all possess anti-biofilm and anti-persister activities (Fig. 2). At a concentration of 8 × MIC, MC-CATH1-3 inhibited *E. coli* biofilm formation by 68.3%, 65.1%, and 56.9%, respectively (Fig. 2*C*), and also inhibited *S. aureus* biofilm formation by 47.3%, 56.6%, and 44%, respectively (Fig. 2*F*). Moreover, MC-CATH1-3 demonstrated the ability to eradicate the preformed biofilms in a concentration-dependent manner. At 8 × MIC, MC-CATH1-3 cleared 77.4%, 75%, and 59.4% of *E. coli* preformed biofilms, respectively (Fig. 2*D*), and also cleared 63.5%, 58.8%, and 74.3% of *S. aureus* preformed biofilms, respectively (Fig. 2*G*). Persisters are dormant cells that tolerate antibiotic treatment and can contribute to recurrent infection (30). MC-CATH1-3 demonstrated the ability to eradicate persisters, with MC-CATH1 being particularly effective. At 8 × MIC, MC-CATH1 eradicated all *E. coli* persisters, while MC-CATH2 and MC-CATH3 eradicated 60% and 81.6% of *E. coli* persisters, respectively (Fig. 2*E*). Similarly, MC-CATH1-3 removed 74%, 72.5%, and 81.1% of *S. aureus* persisters at 8 × MIC, respectively (Fig. 2*H*). Together, these data indicate activity of MC-CATHs against both biofilms and persister cells.

Since most AMPs exert bactericidal effects by targeting bacterial membranes, they may also perturb eukaryotic cell membranes. Therefore, cytotoxicity and hemolysis of MC-CATHs were evaluated. MC-CATH1-3 displayed concentration-dependent cytotoxicity toward HEK293, RAW264.7, and HepG2 cells (Fig. S3, *A*–*C*). Among them, MC-CATH2 showed the strongest cytotoxicity. In hemolysis assays, MC-CATH2 again exhibited the highest activity (10.5% hemolysis at 64 μM), compared to 5.7% for MC-CATH1 and 1.1% for MC-CATH3 (Fig. S3*D*). Among the three peptides, MC-CATH3 exhibited the lowest hemolytic activity, possibly due to its lower hydrophobicity.

### MC-CATHs exhibit different membrane disruption capabilities

**Figure 3:**
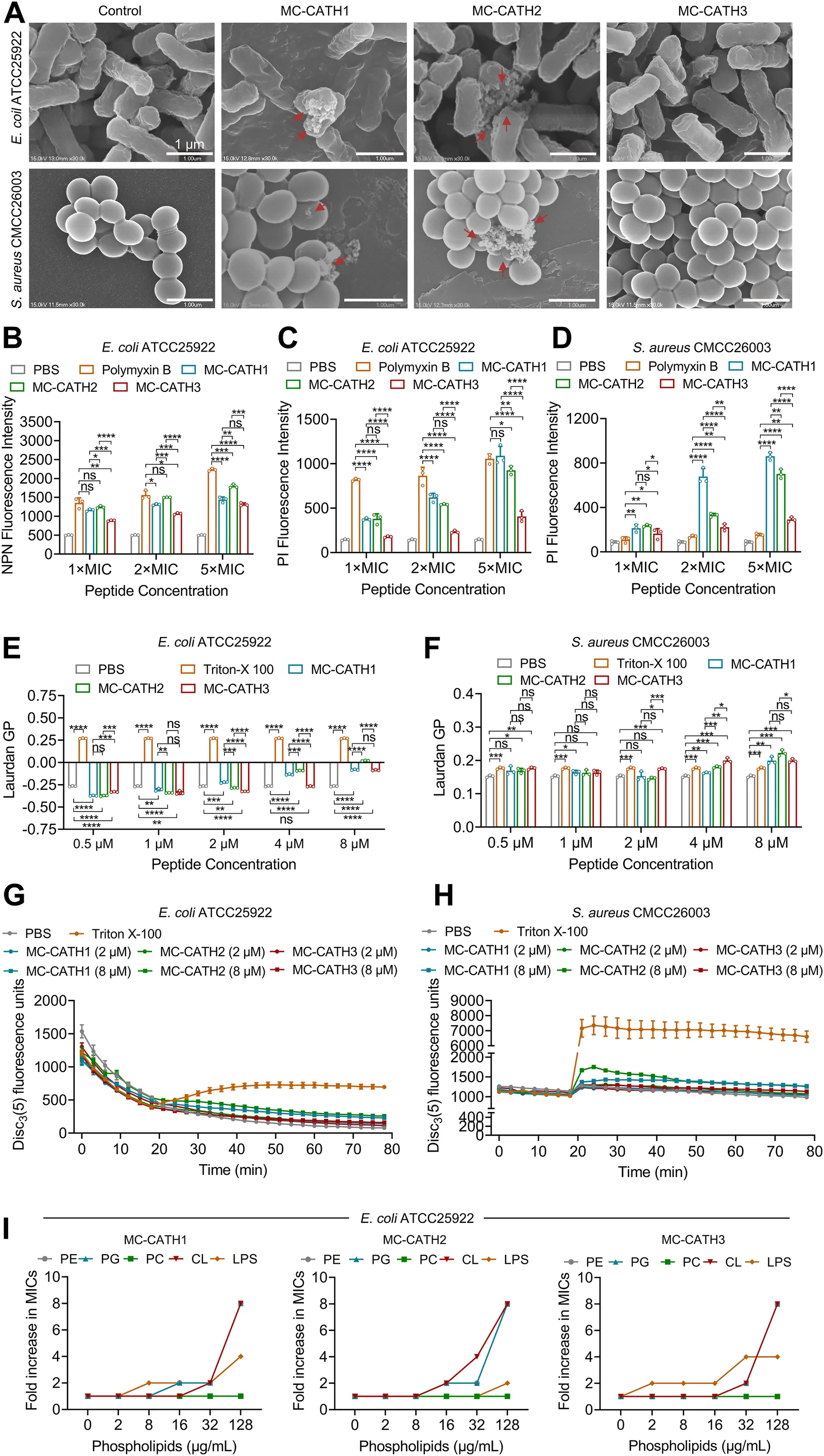
**Membrane-disrupting effects of MC-CATHs.***A*, Scanning electron microscopy (SEM) images of *E. coli* ATCC25922 and *S. aureus* CMCC26003 following exposure to MC-CATHs (5 × MIC) for 2 h. Scar bars: 1 μm. *B*, Effect of MC-CATHs on the outer membrane permeability of *E. coli*, as assessed by NPN fluorescence. Bacterial suspensions were treated with MC-CATHs at 1 × -5 × MIC in the presence of 25 μM NPN, with polymyxin *B* serving as a positive control. *C*-*D*, Inner membrane permeabilization of *E. coli* (*C*) and *S. aureus* (*D*) assessed by propidium iodide (PI) uptake. Bacterial suspensions were treated with MC-CATHs at 1 × -5 × MIC in the presence of 25 μg/ml PI. *E*–*F*, Laurdan GP analysis of membrane fluidity in *E. coli* (*E*) and *S. aureus* (*F*) following MC-CATHs treatment. Bacterial suspensions were treated with MC-CATHs at 0.5 to 8 μM in the presence of 10 μM Laurdan GP, with 1% Triton X-100 serving as a positive control. *G*–*H*, Membrane potential dissipation in *E. coli* (*G*) and *S. aureus* (*H*) measured using Disc_3_(5). Peptides were added after probe stabilization, and fluorescence was monitored over 60 min (excitation/emission: 622/670 nm). Bacterial suspensions were treated with MC-CATHs at 2 μM and 8 μM in the presence of 10 μg/ml Disc_3_(5), with 1% Triton X-100 serving as a positive control. *I*, MICs of MC-CATH peptides against *E. coli* in the presence of exogenous membrane lipids (PE, PG, PC, CL, LPS). Lipids were added to the culture medium at final concentrations ranging from 0 to 128 μg/ml. All experiments were performed in triplicate, and results are presented as mean ± SD. Statistical significance was determined by unpaired *t* test (ns: not significant; ∗*p* < 0.05; ∗∗*p* < 0.01; ∗∗∗*p* < 0.001; ∗∗∗∗*p* < 0.0001).

Cationic AMPs are generally known to kill bacteria primarily by disrupting bacterial cell membranes (31, 32). Because membrane perturbation is also closely related to AMP cytotoxicity, we first investigated the membrane-perturbing effects of MC-CATH1-3. Scanning electron microscopy (SEM) imaging revealed that MC-CATH1 and MC-CATH2 caused marked morphological damage to *E. coli* and *S. aureus*, whereas MC-CATH3 induced negligible surface alterations (Fig. 3*A*). Using the hydrophobic probe NPN, we observed that all three peptides increased outer membrane permeability of *E. coli* in a dose-dependent manner, but MC-CATH3 was consistently weaker than MC-CATH1 and MC-CATH2 (Fig. 3*B*). Similarly, propidium iodide assays showed that MC-CATH3 exerted substantially weaker effects on membrane permeabilization than MC-CATH1 and MC-CATH2 in both *E. coli* and *S. aureus* over the concentration range tested. (Fig. 3, *C* and *D*).

Insertion of AMPs into lipid bilayers typically alters membrane fluidity, leading to leakage of intracellular components and cell death (33, 34). Laurdan GP analysis demonstrated that MC-CATH1-3 reduced the membrane fluidity of both *E. coli* and *S. aureus* in a concentration-dependent manner, with MC-CATH3 again showing the weakest effect (Fig. 3, *E* and *F*). Because disruption of membrane integrity can impair proton motive force (PMF) and thereby disturb bacterial metabolism, we further measured membrane potential using the probe Disc_3_(5). The results indicated that although all three peptides dissipated membrane potential, the effect of MC-CATH3 was markedly weaker than that of MC-CATH1 and MC-CATH2 at equivalent concentrations (Fig. 3, *G* and *H*). Together, these data show that MC-CATH1 and MC-CATH2 induce stronger membrane perturbation than MC-CATH3, consistent with the lower hemolytic activity observed for MC-CATH3.

To identify potential membrane-binding targets of MC-CATH1-3, representative bacterial membrane lipids were exogenously supplemented into the culture medium, followed by evaluation of peptide antibacterial activities. The addition of lipopolysaccharide (LPS) (Fig. 3*I*) markedly reduced antibacterial potency, increasing MIC by up to fourfold. Likewise, stepwise increases in the concentrations of phosphatidylethanolamine (PE), phosphatidylglycerol (PG), and cardiolipin (CL) led to a progressive, dose-dependent decrease in peptide activity, whereas phosphatidylcholine (PC) exerted no detectable influence (Fig. 3*I*). Given that LPS, PG, and CL are anionic in nature, while PC is zwitterionic, these results suggest that MC-CATH1-3 preferentially associate with negatively charged lipid components of bacterial membranes through electrostatic interactions. Among the tested lipids, LPS showed the strongest inhibitory effect on peptide activity, and MC-CATH3 appeared more affected by LPS than the other two peptides.

### MC-CATH3 remains preferentially at the lipid–water interface with reduced insertion into the bilayer

**Figure 4:**
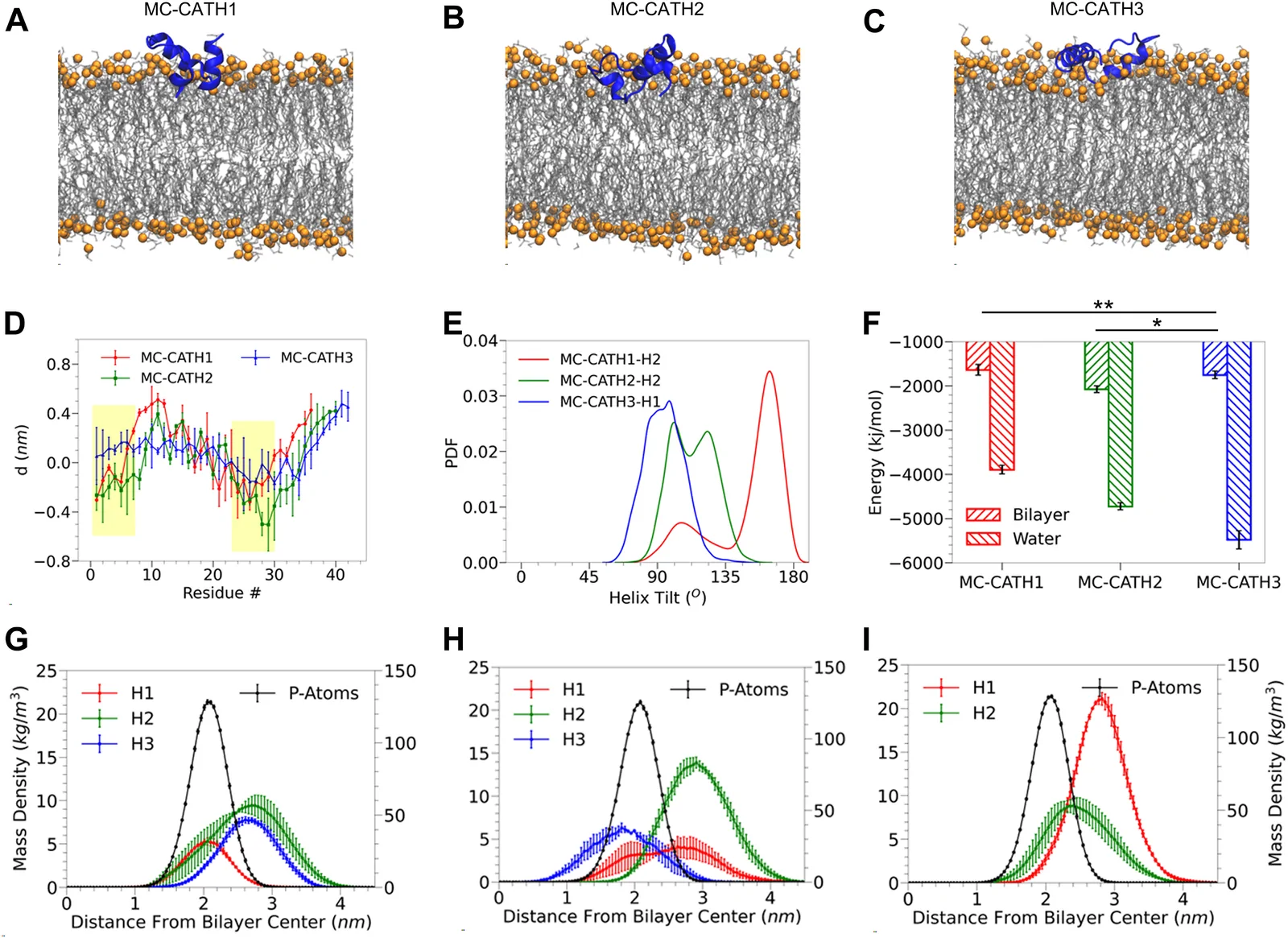
**Molecular dynamics analysis of MC-CATH interactions with lipid bilayers.***A*–*C*, Representative snapshots of MC-CATH1 (*A*), MC-CATH2 (*B*), and MC-CATH3 (*C*) bound to the bilayer. Peptides are shown in *blue*, P atoms of lipid headgroups in *orange*, and lipid tails in *gray*. *D*, Residue-wise insertion depths relative to the bilayer center. *Yellow* regions highlight deeper insertion for MC-CATH1/2 compared to MC-CATH3. *E*, Probability density functions (PDFs) of tilt angles for the longest α-helix of each peptide. *F*, Nonbonded interaction energies between peptides and bilayer or water (200–500 ns). Peptide-bilayer interactions were comparable, while peptide-water interactions differed significantly, increasing from MC-CATH1 to MC-CATH3. *G*–*I*, Mass density profiles of helices relative to lipid P atoms for MC-CATH1 (*G*), MC-CATH2 (*H*), and MC-CATH3 (*I*). Data are shown as mean ± SD; statistical significance determined by unpaired *t* test (∗*p* < 0.05; ∗∗*p* < 0.01).

All-atom molecular dynamics (MD) simulations were conducted to explore the interactions between MC-CATH1-3 and lipid bilayers. Root-mean-square deviation (RMSD) analysis revealed that the three peptides reached equilibrium after approximately 100 to 200 ns (Fig. S4), and all subsequent analyses were based on trajectories collected beyond 200 ns. Representative snapshots from the trajectories (Fig. 4, *A*–*C*) showed that all three peptides bound to the bilayer but exhibited marked differences in insertion depth and orientation. To quantify penetration, residue-wise insertion depths were calculated (Fig. 4*D*). MC-CATH3 remained near the P-atom layer (∼0 Å), whereas MC-CATH1 and MC-CATH2 displayed deeper insertion (∼-0.4 Å). Notably, residues Arg28, Leu29, and Phe30 of MC-CATH2 (within helix-3, Fig. 1*A*) reached depths of ∼ -0.6 Å. Mass density profiles (Fig. 4, *G*–*I*) corroborated these findings: helix-3 of MC-CATH2 penetrated deepest, followed by MC-CATH1, while both helices of MC-CATH3 were localized above the P-atom layer. The peptides also differed in orientation. The longest helix of MC-CATH1 (helix-2, Fig. 1*A*) was generally inserted nearly perpendicular to the bilayer surface, whereas MC-CATH2’s helix-2 adopted two orientations—moderately tilted (∼130°) or nearly parallel (∼100°). In contrast, the longest helix of MC-CATH3 (helix-1, Fig. 1*A*) was almost completely parallel to the bilayer (Fig. 4*E*). Nonbonded interaction energies were next evaluated (Fig. 4*F*; Figs. S4 and S5; Table S1). Peptide-bilayer interaction energies were comparable across the three peptides, but peptide-water interaction energies showed a significant trend of MC-CATH1 < MC-CATH2 < MC-CATH3. These results suggest that MC-CATH3 exhibits stronger interactions with water and preferentially resides at the lipid-water interface, unlike MC-CATH1 and MC-CATH2, which penetrate more deeply into the bilayer. Overall, compared with MC-CATH1 and MC-CATH2, MC-CATH3 showed shallower insertion, a more parallel orientation relative to the bilayer, and stronger interactions with water.

### Other potential antimicrobial mechanisms of MC-CATHs

**Figure 5:**
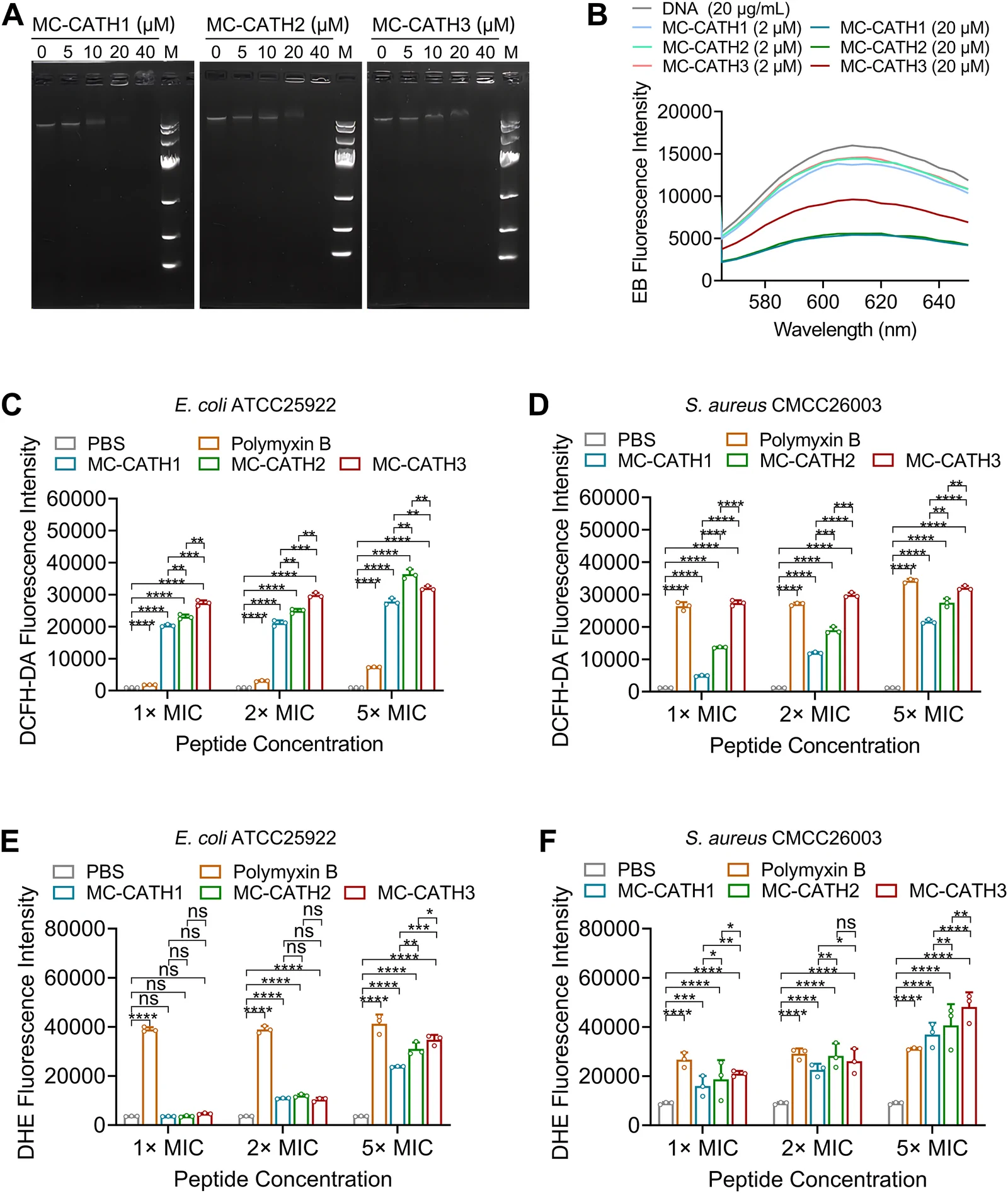
**DNA-binding capacity and ROS induction by MC-CATHs.***A*, Agarose gel retardation assay showing the interaction between bacterial genomic DNA (20 μg/ml) and MC-CATHs (0–40 μM). M, DNA marker. *B*, Ethidium bromide (EB) displacement assay showing fluorescence emission spectra (565–650 nm) of EB-DNA complexes upon titration with MC-CATHs. A decrease in fluorescence intensity reflects EB displacement. *C*–*D*, Intracellular ROS levels in *E. coli* ATCC25922 (*C*) and *S. aureus* CMCC26003 (*D*) following peptide treatment at indicated concentrations, measured using DCFH-DA fluorescence. Polymyxin B and PBS served as controls. *E*–*F*, Intracellular superoxide anion levels in *E. coli* ATCC25922 (*E*) and *S. aureus* CMCC26003 (*F*) following peptide treatment at indicated concentrations, measured using dihydroethidium (DHE) fluorescence. Polymyxin B and PBS served as controls. All experiments were performed in triplicate, and results are presented as mean ± SD. Statistical significance was determined by unpaired *t* test (∗*p* < 0.05; ∗∗*p* < 0.01; ∗∗∗*p* < 0.001; ∗∗∗∗*p* < 0.0001).

MC-CATH3 exhibited markedly weaker membrane-disrupting activity than MC-CATH1 and MC-CATH2, yet retained comparable antimicrobial efficacy, prompting us to examine whether additional mechanisms contribute to its antibacterial activity. Considering that some AMPs have been reported to bind genomic DNA and thereby inhibit replication or transcription, leading to bacterial death (14), we evaluated the DNA-binding capacity of MC-CATH1-3 using agarose gel retardation and ethidium bromide (EB)-DNA competition assays. In the gel retardation assay, increasing peptide concentrations progressively retarded the migration of bacterial genomic DNA, indicating the formation of stable peptide-DNA complexes (Fig. 5*A*). Notably, MC-CATH1 achieved complex formation at lower concentrations (20 μM) than MC-CATH2 and MC-CATH3 (40 μM), reflecting stronger DNA-binding affinity. These findings were corroborated by EB displacement assays. All three peptides reduced EB-DNA fluorescence in a concentration-dependent manner (Fig. 5*B*), but MC-CATH3 displayed significantly weaker competitive binding. At 20 μM, its displacement efficiency was only about half that of MC-CATH1 and MC-CATH2, indicating relatively weaker DNA-binding activity.

Given the weak membrane-disrupting and DNA-binding ability of MC-CATH3, we next asked whether it might induce other intracellular responses associated with bacterial killing. Previous studies have shown that certain intracellularly targeted antibiotics and AMPs exert bactericidal effects by triggering oxidative stress and ROS accumulation (19). We therefore investigated the ROS-inducing capacity of MC-CATHs (20, 35, 36). Using the DCFH-DA probe, we observed that all three peptides significantly increased intracellular ROS levels in *E. coli* and *S. aureus*. Remarkably, MC-CATH3 induced substantially higher ROS accumulation than MC-CATH1 and MC-CATH2 at 1× and 2× MIC (Fig. 5, *C* and *D*). To further characterize the oxidative stress response, we used the superoxide-sensitive probe Dihydroethidium (DHE) to assess intracellular superoxide generation. The results showed that all three peptides significantly increased DHE fluorescence in a concentration-dependent manner in both *E. coli* and *S. aureus*, indicating the induction of intracellular superoxide accumulation. Notably, at 5× MIC, MC-CATH3 generally elicited higher DHE fluorescence than MC-CATH1 and MC-CATH2, and this trend was observed in both bacterial species (Fig. 5, *E* and *F*). Therefore, in contrast to MC-CATH1 and MC-CATH2, whose antibacterial activities appear to rely more heavily on membrane disruption and DNA binding, oxidative stress induction may contribute more substantially to the antibacterial activity of MC-CATH3.

### Comparative transcriptomics analysis of MC-CATHs reveals distinct antibacterial mechanisms

**Figure 6:**
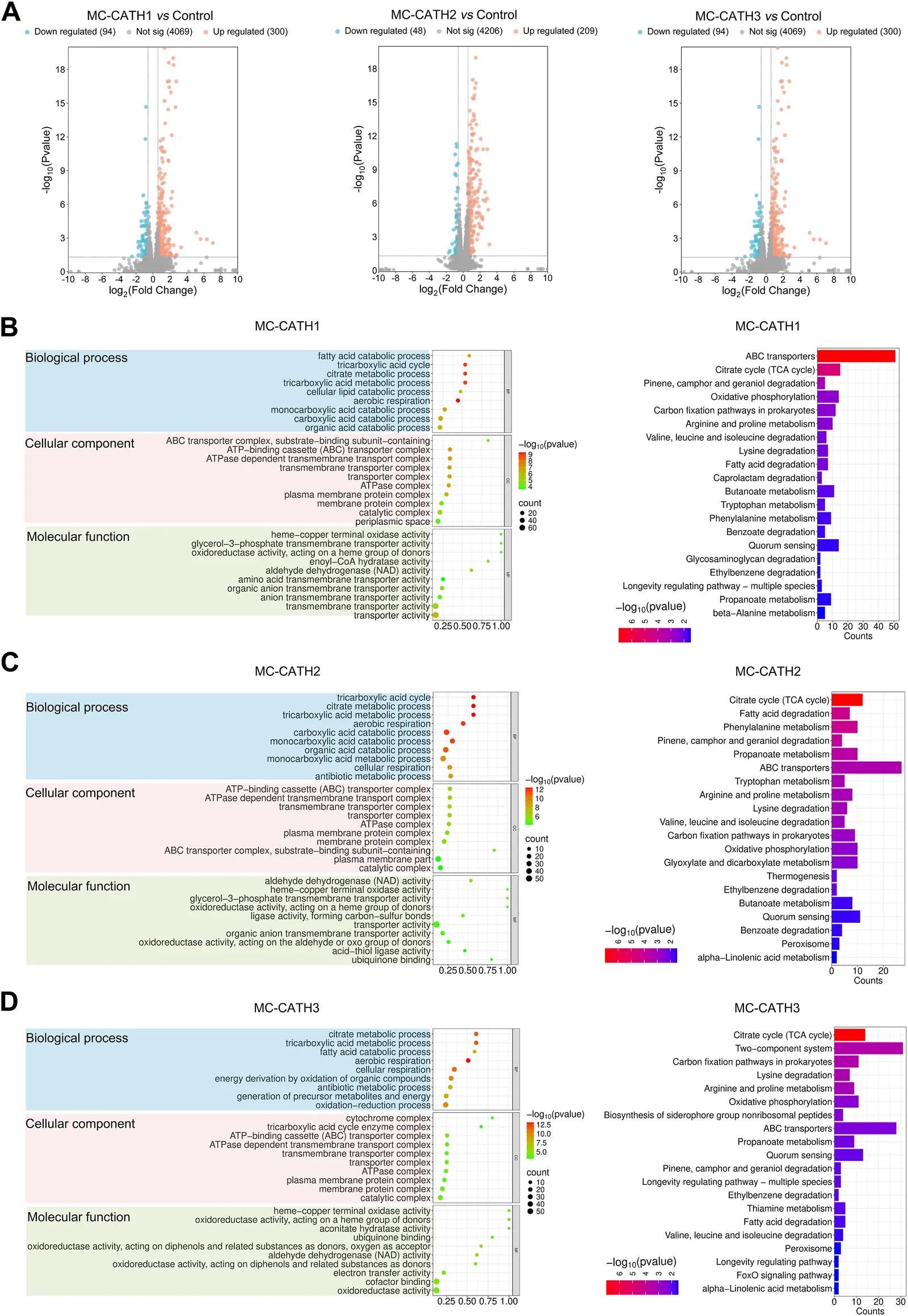
**Transcriptomic and functional characterization of MC-CATHs in *E. col*i ATCC25922.***A*, Volcano plots of differentially expressed genes (DEGs) in *E. coli* ATCC25922 treated with MC-CATH1, MC-CATH2, or MC-CATH3 *versus* control, respectively (*red*: upregulated; *blue*: downregulated; *gray*: not significant). *B*–*D*, Gene Ontology (GO) enrichment analysis and KEGG pathway enrichment analysis of DEGs regulated by MC-CATH1 (*B*), MC-CATH2 (*C*), and MC-CATH3 (*D*), respectively. GO enrichment analysis includes biological process, cellular component, and molecular function categories, and KEGG pathway analysis shows the *top* enriched metabolic and signaling pathways affected by each peptide.

MC-CATH3 provoked significantly stronger intracellular ROS accumulation than MC-CATH1 or MC-CATH2, which prompted further investigation into its underlying mechanism. Transcriptome profiling of *E. coli* ATCC25922 revealed 394 differentially expressed genes (DEGs) for MC-CATH1 (300 up/94 down; Fig. 6*A*), 257 DEGs for MC-CATH2 (209 up/48 down; Fig. 6*A*), and 353 DEGs for MC-CATH3 (286 up/67 down; Fig. 6*A*). GO enrichment across these sets highlighted processes associated with the TCA cycle, aerobic respiration, and oxidative metabolism (Fig. 6, *B*–*D*, left panels). At the cellular-component level, enriched terms included the cytochrome complex, ABC transporter complex, ATPase/transmembrane transporters, and plasma-membrane protein complexes, while molecular functions were enriched for heme-copper oxidase, oxidoreductase, aconitase, electron-transfer, and NAD-dependent dehydrogenase activities (Fig. 6, *B*–*D*, left panels). Notably, MC-CATH1 and MC-CATH2 preferentially impacted transporter-related functions (*e.g.*, amino acid, ion, and glycerol-phosphate transport), whereas MC-CATH3 predominantly influenced oxidoreduction-related pathways, including electron-transfer-associated activities. KEGG enrichment reinforced these trends (Fig. 6, *B*–*D*, right panels), with MC-CATH3 showing stronger engagement of oxidative phosphorylation, quorum sensing, and siderophore biosynthesis. Together, these transcriptional changes are consistent with a stronger perturbation of oxidative metabolism and iron-related pathways.

**Figure 7:**
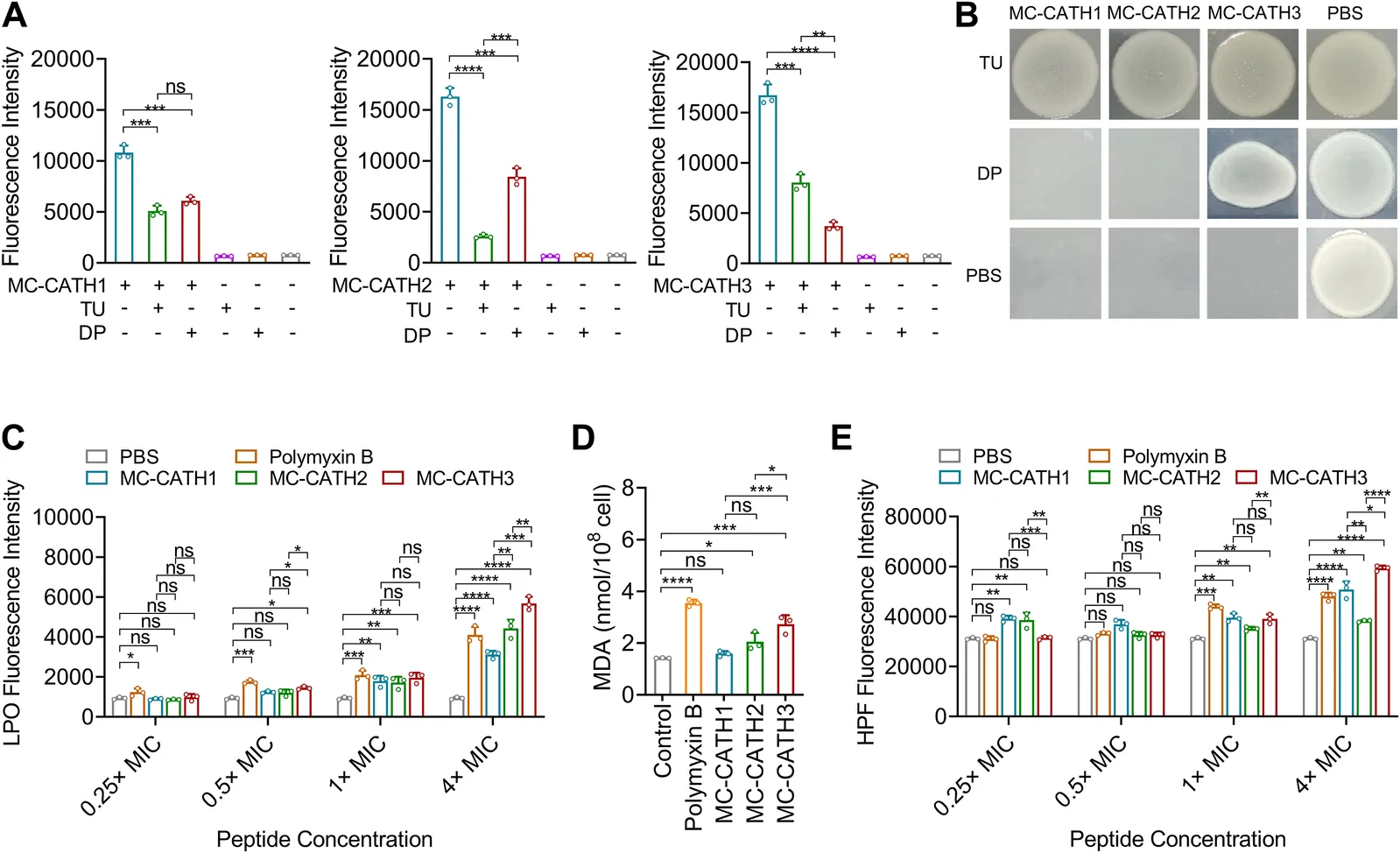
**Oxidative damage contributes to the antibacterial activity of MC-CATHs.***A*, Intracellular ROS assays (DCFH-DA fluorescence) in *E. coli* exposed to MC-CATHs with TU (300 mM) or DP (80 μM). MC-CATHs were applied at 8 μM, and the probe concentration was 10 μM. *B*, Representative colony images showing the effects of TU or DP on *E. coli* survival following MC-CATH treatment. MC-CATHs were applied at a final concentration of 8 μM; DP and TU were used at final concentrations of 80 μM and 300 mM, respectively. *C*, Lipid peroxidation levels in *E. coli* treated with MC-CATH peptides were determined using the lipid peroxidation probe C11-BODIPY581/591. MC-CATHs were applied at 0.25 × -4 × MIC, and the probe concentration was 10 μM. *D*, Malondialdehyde (MDA) levels in *E. coli* following MC-CATHs treatment. MC-CATHs were applied at 5 × MIC. *E*, Hydroxyl and peroxynitrite radicals generation in *E. coli* as a function of MC-CATHs concentration was evaluated using HPF fluorescence intensity. MC-CATHs were applied at 0.25 × -4 × MIC, and the probe concentration was 10 μM. All experiments were performed in triplicate, and data are presented as mean ± SD. Statistical significance was assessed by unpaired *t* test (ns: not significant; ∗*p* < 0.05; ∗∗*p* < 0.01; ∗∗∗*p* < 0.001; ∗∗∗∗*p* < 0.0001).

Both the ROS assay and the transcriptome sequencing suggested that MC-CATH peptides perturb bacterial oxidative metabolism and electron transport, thereby promoting intracellular ROS accumulation. To further investigate the contribution of ROS, two scavengers were used. Thiourea (TU) directly scavenges intracellular ROS, whereas 2,2′-dipyridyl (DP) suppresses ROS generation associated with the Fenton reaction. *E. coli* and *S. aureus* were treated with each peptide in the presence or absence of DP or TU, and intracellular ROS levels were measured using DCFH-DA fluorescence (Fig. 7*A*; Fig. S6*G*). Compared with the untreated controls, all three MC-CATH peptides significantly increased intracellular ROS levels in both *E. coli* and *S. aureus*. Although the magnitudes of the effects of TU and DP were not identical between the two bacterial species, both datasets showed that ROS accumulation was a common change following peptide treatment. In comparison, MC-CATH3 exhibited a more pronounced response to DP, and its bactericidal activity was significantly reduced after DP treatment, particularly in *E. coli*. These results suggest that the antibacterial activity of MC-CATH3 may be more closely associated with an iron-dependent oxidative process.

To further assess the contribution of ROS to the bactericidal activities of the peptides, bacteria were exposed to lethal peptide concentrations in the presence of the ROS scavengers. TU markedly rescued both *E. coli* and *S. aureus* from MC-CATH1-3-induced killing, whereas DP conferred protection only under MC-CATH3 treatment (Fig. 7*B*; Fig. S6*H*). These observations further support a prominent contribution of oxidative stress to MC-CATH3-mediated killing and are consistent with a role for Fe^2+^-associated ROS generation.

Because iron-dependent lipid peroxidation is a defining feature of ferroptosis and has also been proposed in bacteria, we next examined whether MC-CATH3 treatment was associated with increased lipid oxidation (22, 25). To assess the effect of MC-CATH3 on bacterial lipid peroxidation, we measured lipid oxidation levels in peptide-treated *E. coli* and *S. aureus* using the C11-BODIPY581/591 probe and a malondialdehyde (MDA) quantification assay. C11-BODIPY581/591 is a well-established fluorescent probe that detects lipid peroxidation by exhibiting a red-to-green emission shift (590 nm to 510 nm) upon oxidation, owing to its strong lipophilicity, rapid membrane penetration, and high sensitivity to ROS within membranes. Both *E. coli* and *S. aureus* exposed to MC-CATH1-3 showed concentration-dependent increases in lipid peroxidation (Fig. 7*C*; Fig. S6*I*). Notably, MC-CATH2 and MC-CATH3 induced significantly higher oxidation levels (Fig. 7*C*). In *S. aureus*, although MC-CATH3 caused slightly lower lipid peroxidation than MC-CATH2, it remained markedly higher than that induced by MC-CATH1 (Fig. S6*I*). Consistently, MDA quantification revealed substantial increases in MDA levels in both bacterial species following treatment with MC-CATH2 and MC-CATH3 (Fig. 7*D*; Fig. S6*J*). These results indicate that MC-CATH2 and MC-CATH3 are both associated with strong lipid peroxidation, although the upstream oxidative processes may differ.

Hydroxyl radicals are important drivers of lipid peroxidation (37, 38). To evaluate intracellular hydroxyl radical levels, we used the HPF probe, which selectively reacts with hydroxyl and peroxynitrite radicals (Fig. 7*E*; Fig. S6*K*). Both *E. coli* and *S. aureus* exhibited a marked increase in HPF fluorescence following treatment with MC-CATH3, indicating enhanced hydroxyl radical production (Fig. 7*E*; Fig. S6*K*). MC-CATH1 also caused a strong HPF signal but did not promote lipid peroxide accumulation (Fig. 7*C*; Fig. S6*I*), suggesting predominant peroxynitrite generation rather than hydroxyl radical accumulation (Fig. 7*E*; Fig. S6*K*). Conversely, MC-CATH2 strongly promoted lipid peroxidation without increasing HPF fluorescence. Taken together, these data suggest that MC-CATH3-induced antibacterial activity is associated with iron-dependent oxidative stress and lipid peroxidation, consistent with the involvement of a ferroptosis-like process in bacteria.

### MC-CATH3 induces iron-dependent oxidative stress as an antibacterial mechanism

**Figure 8:**
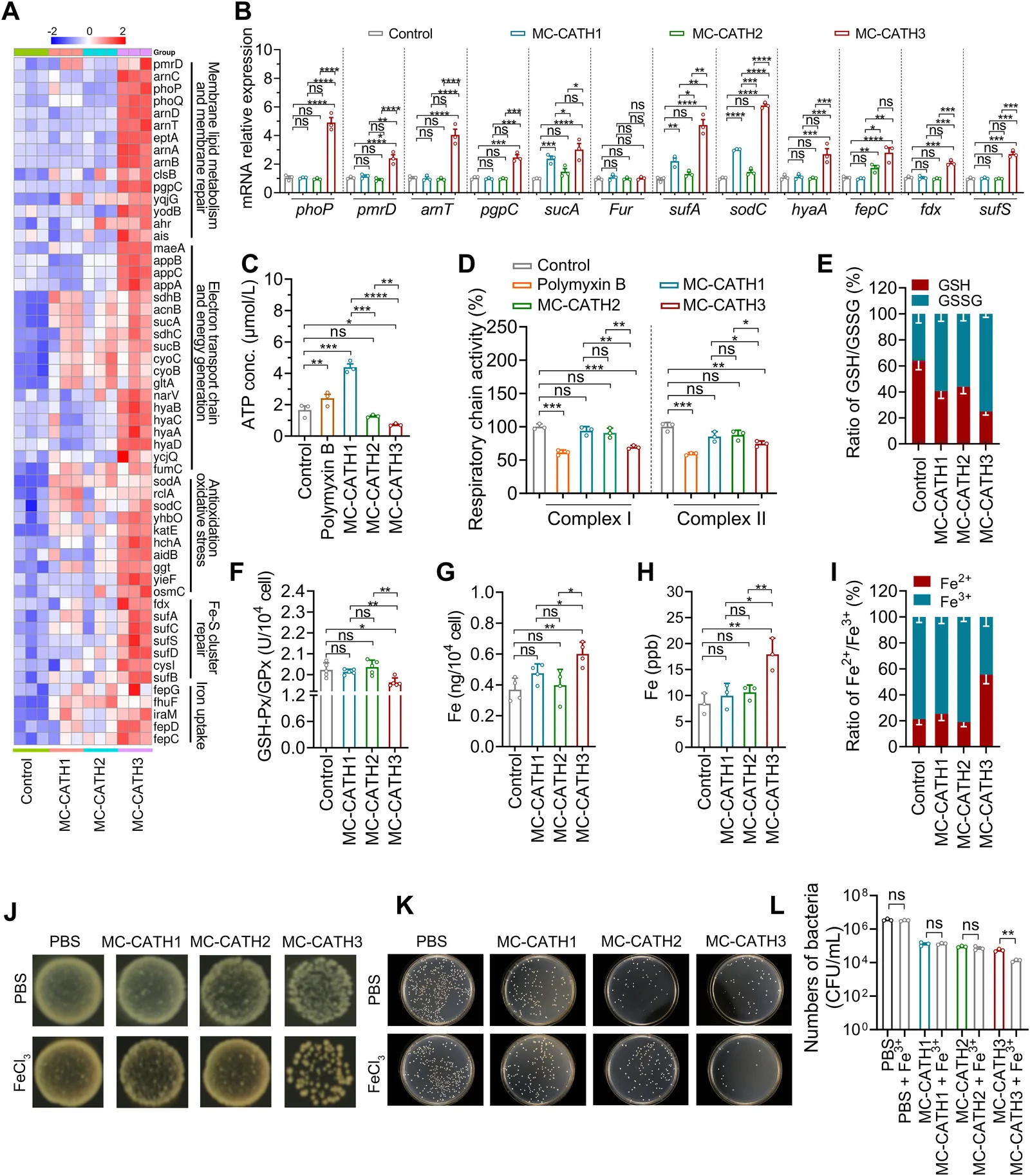
**Mechanistic cascade of MC-CATH3-induced iron-dependent oxidative killing in *E. col*i ATCC25922.***A*, Heatmap of selected DEGs induced by MC-CATH peptides, grouped into membrane lipid metabolism and membrane repair, electron transport/energy generation, oxidative stress response, Fe-S cluster repair, and iron uptake. Gene expression values are shown as row Z-scores across groups; *red* and *blue* indicate relatively *higher* and *lower* expression levels, respectively. *B*, qPCR validation of representative genes in iron metabolism, oxidative-stress response, and membrane pathways. *C*, Intracellular ATP concentration measured in untreated control cells and cells exposed to MC-CATHs. MC-CATHs were applied at 5 × MIC. *D*, Mitochondrial respiratory chain activity determined for complex I and complex II following peptide exposure. MC-CATHs were applied at 5 × MIC. *E*, Cellular redox balance shown as the ratio of reduced glutathione (GSH, *red*) to oxidized glutathione (GSSG, *blue*), expressed as percentage, across the same experimental conditions. MC-CATHs were applied at 5 × MIC. *F*, Glutathione peroxidase (GSH-Px/GPx) enzymatic activity quantified in control and peptide-treated groups. MC-CATHs were applied at 5 × MIC. *G*, Total intracellular iron content measured in each treatment group; values reflect total Fe accumulation under the indicated conditions. MC-CATHs were applied at 5 × MIC. *H*, Total intracellular iron content quantified by inductively coupled plasma mass spectrometry (ICP-MS) in *E. coli* following MC-CATH treatment. MC-CATHs were applied at 5 × MIC. *I*, Distribution of ferrous (Fe^2+^, *red*) and ferric (Fe^3+^, *blue*) iron species shown as percentage (%), indicating changes in intracellular iron redox state after peptide treatment. MC-CATHs were applied at 5 × MIC. *J*, Effect of exogenous addition of FeCl_3_ on the antibacterial activity of MC-CATHs. *K*, Representative dilution plating assay showing the survival of *E. coli* after MC-CATH treatment in the presence or absence of exogenous FeCl_3_. *L*, Quantification of viable bacteria from the dilution plating assay under the indicated treatment conditions. For *J–L*, MC-CATHs were applied at 2 × MIC, and FeCl_3_ was added at a final concentration of 32 μM. All experiments were performed in triplicate, and data are presented as mean ± SD. Statistical significance was assessed by unpaired *t* test (ns: not significant; ∗*p* < 0.05; ∗∗*p* < 0.01; ∗∗∗*p* < 0.001; ∗∗∗∗*p* < 0.0001).

At the transcriptomic level, MC-CATH1-3 triggered extensive gene-expression reprogramming in *E. coli* ATCC25922, with MC-CATH3 exerting the most pronounced overall effect. The selected DEGs shown in Figure 8*A* were mainly associated with membrane lipid metabolism and membrane repair, electron transport/energy generation, oxidative stress response, Fe-S cluster repair, and iron uptake. It is noteworthy that, despite their stronger membrane-disruptive activity, MC-CATH1 and MC-CATH2 did not show marked upregulation of genes related to membrane lipid metabolism and membrane repair at the 2-h sampling point used in this study. One possible explanation is that their rapid and severe membrane damage may have impaired the establishment of a subsequent adaptive transcriptional response. In contrast, the relatively weaker direct membrane-disruptive effect of MC-CATH3 may induce a membrane stress state that remains transcriptionally responsive and is therefore detectable at this time point. qPCR analyses confirmed these observations: MC-CATH3 strongly up-regulated membrane-stress and lipid-modification genes (*phoP*, *pmrD*, *arnT*), indicating disruption of membrane integrity, while also inducing energy-metabolism genes (*sucA*, *sdhB*, *hyaA*), Fe-S cluster repair genes (*sufA*, *sufS*), and the iron-uptake regulator *fepC* (Fig. 8*B*). Together, these transcriptional responses are consistent with membrane stress accompanied by perturbation of cellular energy metabolism, Fe-S homeostasis, and iron-related pathways under MC-CATH3 treatment.

To examine whether these transcriptomic changes were accompanied by altered energy metabolism, we assessed cellular energy status. MC-CATH3 treatment was associated with upregulation of lipid-synthesis/repair pathways and tricarboxylic-acid-cycle (TCA) genes, suggesting an adaptive metabolic response. Nevertheless, intracellular ATP levels were markedly reduced (Fig. 8*C*), indicating impaired energy production. To further assess whether this energy deficit was associated with respiratory dysfunction, we measured the activities of Complex I (NADH dehydrogenase) and Complex II (succinate dehydrogenase). MC-CATH3 markedly reduced the catalytic activities of both complexes (Fig. 8*D*), consistent with the ATP-depletion pattern. Given that these complexes contain multiple Fe-S clusters as electron-transfer centers, their reduced activity may be associated with oxidative damage to Fe-S-containing components, which could destabilize electron flow and favor ROS generation. In agreement, transcriptomic data showed coordinated upregulation of genes involved in energy metabolism and Fe-S cluster assembly or repair (*sucA*, *sdhB*, *hyaA*, *sufA*, *sufS*, *cysI*) (Fig. 8, *A* and *B*). These findings suggest that MC-CATH3 treatment is associated with concurrent respiratory dysfunction, ATP depletion, and induction of Fe-S repair-related responses.

We next examined cellular redox homeostasis. Reduced glutathione (GSH) is a major non-enzymatic antioxidant that scavenges ROS and helps maintain a reducing intracellular environment. When GSH is consumed, it is oxidized to glutathione disulfide (GSSG); thus, the GSH/GSSG ratio reflects cellular redox capacity. MC-CATH3 treatment markedly decreased this ratio while increasing GSSG levels (Fig. 8*E*), indicating a shift toward a more oxidizing intracellular environment. These findings align with the decreased activities of Complex I/II and the transcriptional induction of Fe-S repair genes (Fig. 8, *A*, *B* and *D*). The resulting oxidative stress may contribute to Fe-S damage, while the upregulation of *sufA*/*sufS* is consistent with a compensatory repair response. Given the role of the glutathione system in ROS detoxification, including the reduction of lipid hydroperoxides, we next assessed GPx activity. MC-CATH3 appeared to reduce GPx activity, whereas MC-CATH2 increased it (Fig. 8*F*). Combined with lipid-peroxide measurements (Fig. 7*C*), these data indicate that the peptides differ in their downstream effects on bacterial redox balance and lipid oxidation. In particular, the lower GPx activity observed under MC-CATH3 treatment may contribute to less efficient detoxification of lipid peroxides and thereby favor oxidative lipid damage. The molecular basis for the increased GPx activity under MC-CATH2 treatment remains unclear.

Given that iron-dependent oxidative reactions can amplify lipid peroxidation, we further investigated whether MC-CATH3 affects iron homeostasis. Fe^3+^ generally exists as a storage form, while Fe^2+^ is more catalytically active and can promote hydroxyl-radical generation through Fenton chemistry. MC-CATH3 markedly increased total intracellular iron levels (Fig. 8, *G* and *H*) and elevated the Fe^2+^/Fe^3+^ ratio (Fig. 8*I*), suggesting expansion of the labile and more reduced iron pool. To further assess the contribution of iron, exogenous FeCl_3_ was added under sub-bactericidal conditions. Iron supplementation markedly enhanced MC-CATH3 toxicity (Fig. 8, *J* and *K*), supporting a relationship between iron availability and bactericidal potency. Consistently, transcriptomic data showed upregulation of multiple genes associated with iron uptake and Fe-S cluster repair, including *fepC*, *sufD*, *sufC*, *sufB*, *sufA*, and *fdx* (Fig. 8, *A* and *B*). The induction of *fepC* is consistent with increased iron acquisition, whereas the coordinated activation of Fe-S-related genes may reflect adaptation to oxidative stress and Fe-S damage. Together, these findings suggest that iron dysregulation contributes to MC-CATH3-mediated killing and may participate in a ferroptosis-like death program.

Collectively, these data indicate that MC-CATH3 triggers membrane stress together with respiratory, redox, and iron dyshomeostasis, leading to iron-dependent oxidative damage accompanied by lipid peroxidation, a pattern reminiscent of ferroptosis-like features in bacteria. This mechanism differs from the more membrane-disruptive and DNA-binding-associated activities observed for MC-CATH1/2. Notably, MC-CATH3 elicited similar metabolic responses in *S. aureus*, including pronounced ATP depletion, inhibition of respiratory-complex I/II activity, decreased GSH/GSSG ratio, reduced GPx activity, and increased total iron content and Fe^2+^/Fe^3+^ ratio (Fig. S7, *A*–*J*), suggesting that this oxidative response may also occur in Gram-positive bacteria.

### MC-CATHs accelerate healing of *S. Aureus*-infected wounds *in vivo*

**Figure 9:**
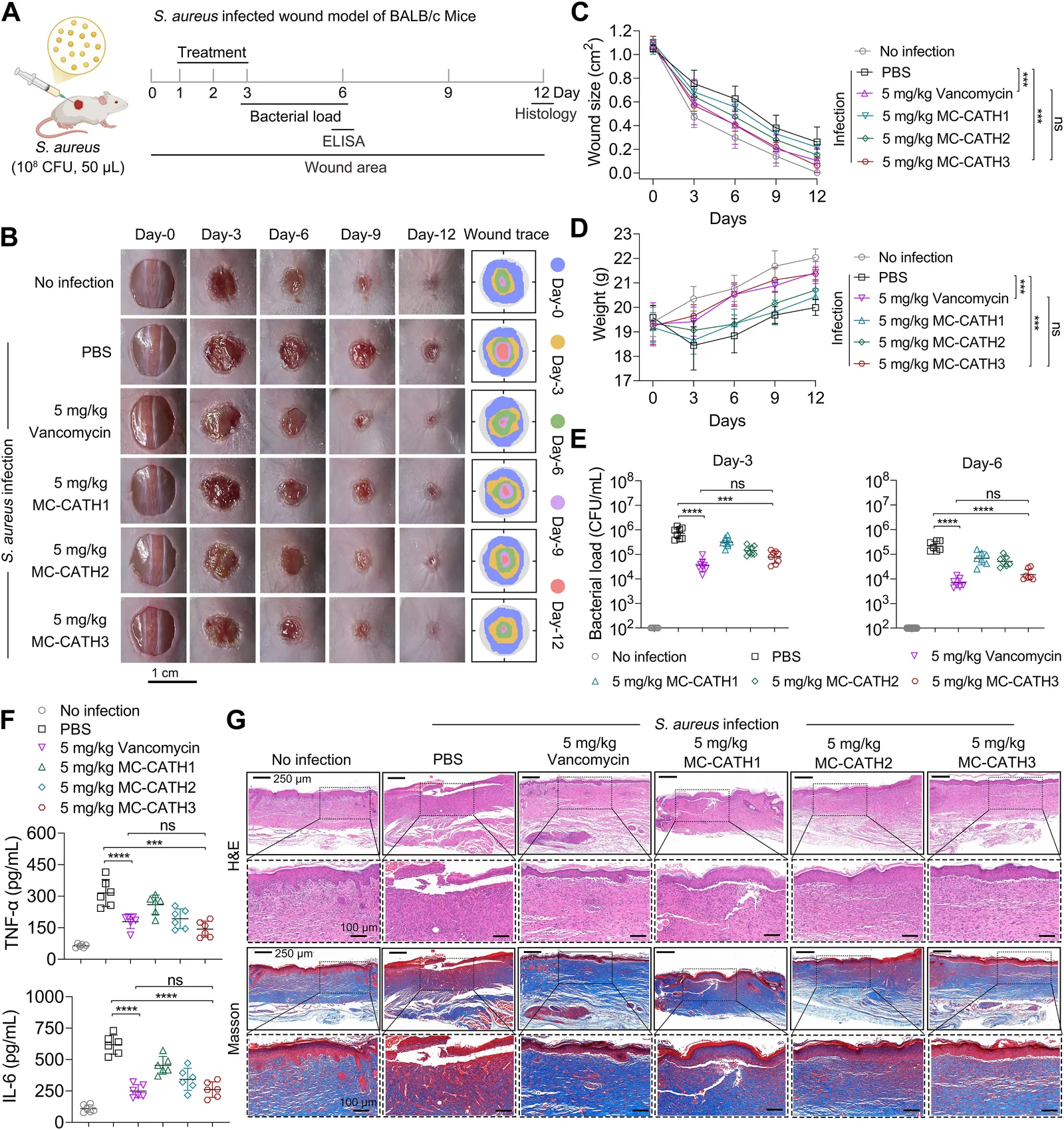
**MC-CATHs promote healing of *S. aureus* infected skin wounds in mice.***A*, Schematic of the murine excisional wound infection model (*n* = 8 per group). Full-thickness dorsal wounds were created in BALB/c mice and inoculated with *S. aureus* at day 0. Infected mice were treated once daily with PBS, vancomycin, or MC-CATHs for the indicated period, whereas a non-infected wound group served as a sham control. Wound tissues were collected at the indicated time points for bacterial enumeration, ELISA, and histological analysis. *B*, Representative photographs of wounds from each group, including uninfected control, PBS, vancomycin 5 mg/kg, MC-CATH1 5 mg/kg, MC-CATH2 5 mg/kg, and MC-CATH3 5 mg/kg, on days 0, 3, 6, 9, and 12. Colored wound traces adjacent to the day-12 images delineate the sequential wound boundaries used for quantitative analysis of wound area. Vancomycin and PBS served as positive and negative controls, respectively. Scale bar, 1 cm. *C*, Quantification of wound area at the indicated days after infection. *D*, Body weight changes of mice in each group during the course of treatment. *E*, Bacterial burden in wound tissues at days 3 and 6. *F*, Concentrations of TNF-α and IL-6 in sera determined by ELISA on day 6. *G*, Representative H&E and Masson’s staining of wound sections collected on day 12, showing re-epithelialization, granulation tissue formation, inflammatory cell infiltration, and collagen deposition in the different treatment groups. Scale bars, 250 μm or 100 μm. Data are presented as mean ± SD. Statistical significance was assessed by unpaired *t* test (ns, not significant; ∗∗∗*p* < 0.001; ∗∗∗∗*p* < 0.0001).

To evaluate the *in vivo* anti-infective activities of MC-CATH peptides, we established a murine excisional wound model infected with *S. aureus*. Full-thickness dorsal wounds were inoculated with *S. aureus* and treated with PBS, vancomycin, or MC-CATHs, with non-infected wounds serving as sham controls (Fig. 9*A*). Macroscopic monitoring of wound closure showed that all three MC-CATH peptides significantly accelerated wound healing compared with PBS. Wounds in the PBS group remained large and poorly contracted over the 12-days observation period, whereas vancomycin- and MC-CATH–treated wounds progressively shrank, with nearly complete closure by day 12 (Fig. 9, *B* and *C*). Among the peptides, MC-CATH3 produced the most pronounced improvement in wound contraction, with closure dynamics closely matching those of the vancomycin group, indicating favorable *in vivo* efficacy. Body weight monitoring further supported the favorable profile of MC-CATHs. While PBS-treated infected mice exhibited a decline or slower recovery in body weight, animals treated with vancomycin or MC-CATHs, especially MC-CATH3, maintained or recovered body weight more rapidly, consistent with improved infection control and the absence of obvious systemic intolerance under the conditions tested (Fig. 9*D*).

Consistent with the macroscopic healing outcomes, bacterial load quantification demonstrated that all three MC-CATH peptides significantly reduced *S. aureus* burden at the wound site on days 3 and 6. The reductions in CFU in the MC-CATH groups were markedly greater than in the PBS group, with MC-CATH3 achieving bacterial clearance comparable to vancomycin (Fig. 9*E*). These data indicate that MC-CATHs not only accelerate wound closure but also effectively eradicate bacteria *in vivo*. Inflammatory cytokine measurements revealed that MC-CATHs treatment also modulated the local inflammatory milieu. Infection markedly increased TNF-α and IL-6 levels in PBS-treated wounds, reflecting a strong pro-inflammatory response. In contrast, vancomycin and MC-CATHs significantly lowered the concentrations of both cytokines, with MC-CATH3 again showing the strongest suppression (Fig. 9*F*). This suggests that MC-CATHs alleviate excessive inflammation while controlling infection, thereby creating a more favorable environment for tissue repair. Histological analyses provided further mechanistic support for the beneficial effects of MC-CATHs on wound healing (Fig. 9*G*). H&E staining showed that PBS-treated infected wounds were characterized by incomplete re-epithelialization, disorganized granulation tissue, and dense inflammatory cell infiltration. In comparison, vancomycin- and MC-CATH3–treated wounds exhibited more continuous epidermal coverage, thicker and more mature granulation tissue, and reduced inflammatory infiltrates (Fig. 9*G*). Masson’s staining revealed sparse and disordered collagen fibers in PBS-treated wounds, whereas MC-CATH treatment, particularly with MC-CATH3, led to abundant, well-organized collagen deposition resembling that observed in vancomycin-treated and uninfected control skin (Fig. 9*G*). Together, these data show that MC-CATHs, particularly MC-CATH3, promote healing of *S. aureus*-infected cutaneous wounds *in vivo*, accompanied by reduced bacterial burden, attenuated local inflammation, and improved tissue repair.

## Discussion

AMPs are promising candidates for combating drug-resistant bacteria because their cationic and amphiphilic properties enable rapid bacterial killing and make resistance development more difficult than with many conventional antibiotics (10, 39). Among them, vertebrate cathelicidins are of particular interest because, in addition to direct antimicrobial activity, they often promote chemotaxis, wound healing, and angiogenesis while exhibiting relatively low hemolytic and cytotoxic effects (40). However, strong membrane-disruptive activity can also increase mammalian cytotoxicity (41, 42), highlighting the value of AMPs with effective non-lytic antibacterial mechanisms (16).

Consistent with these general features, MC-CATH1-3 from *M. chinensis* are cationic peptides (+6 to +7) that adopt amphipathic α-helical conformations, a structural organization that favors initial membrane interaction (Table 1, Fig. 1). All three peptides exhibited broad-spectrum antibacterial activity against both Gram-negative and Gram-positive bacteria, with MICs ranging from 0.78 to 75 μg/ml, together with rapid bactericidal effects (Table 2, Fig. 2). Moreover, AMPs with homologous expression often exhibit synergistic antibacterial effects (43). When combined, the three homologous peptides displayed pronounced synergistic effects against *Acinetobacter baumannii* and *S. aureus* (FICI ≤ 0.5), suggesting that cooperative interactions enhance their overall antibacterial efficacy (Fig. S2). Additionally, all three peptides effectively inhibited biofilm formation and targeted dormant bacterial cells (Fig. 2). In terms of cytotoxicity, they showed concentration-dependent effects, with MC-CATH2 being the most cytotoxic and MC-CATH3 the least (Fig. S3), indicating that MC-CATH3 maintains antibacterial potency while exhibiting higher safety toward mammalian cells.

To elucidate their mechanism of action, we further examined the effects of the peptides on bacterial membranes. MC-CATH1 and MC-CATH2 caused pronounced membrane disruption, particularly severe damage to the outer membrane of Gram-negative bacteria, whereas MC-CATH3 displayed weaker membrane disruption, exerting minimal effects on the inner membrane under identical conditions (Fig. 3). Molecular dynamics simulations revealed that MC-CATH3 binds only superficially to the lipid bilayer, remaining stabilized mainly through interfacial interactions with water molecules rather than penetrating deeply into the membrane (Fig. 4). This suggests that MC-CATH3 may act through a less lytic mechanism distinct from classical membrane-permeabilizing AMPs. Competitive lipid-binding assays further showed that all three peptides preferentially interact with bacterial anionic lipids (LPS, PG, CL) over zwitterionic/neutral lipids (*e*.*g*., PC, PE). Notably, MC-CATH3 exhibited the highest sensitivity toward LPS binding, suggesting a predominant surface-localized, interfacial mode of action (Fig. 3).

Cationic AMPs can also bind DNA through electrostatic interactions with the negatively charged phosphate backbone, thereby contributing to antibacterial activity (44). Consistent with this, all three peptides were capable of binding to DNA, with MC-CATH1 showing the strongest affinity and MC-CATH3 the weakest (Fig. 5). Notably, despite its weaker DNA-binding capacity, MC-CATH3 induced substantial ROS accumulation even at low concentrations (1 × MIC) (Fig. 5), suggesting the involvement of a distinct antimicrobial pathway. To further assess the contribution of ROS to peptide-mediated antibacterial activity, the hydroxyl radical scavenger thiourea (TU) and iron chelator 2,2′-dipyridyl (DP) were employed. In both *E. coli* and *S. aureus*, all three peptides induced intracellular ROS accumulation, and these oxidant signals were partially reduced by TU or DP treatment (Fig. 7, Fig. S6), supporting a general involvement of oxidative stress in their antibacterial effects. Although the overall ROS responses varied depending on the peptide and bacterial species, MC-CATH3 consistently showed a more pronounced reduction in ROS levels in the presence of DP, particularly in *E. coli*. Spot assays further showed that DP markedly attenuated the bactericidal activity of MC-CATH3 against both *E. coli* and *S. aureus* (Fig. 7, Fig. S6). Together, these findings suggest that, in addition to general ROS accumulation, MC-CATH3-mediated killing may involve a greater contribution from DP-sensitive oxidative processes, potentially including hydroxyl radicals generated through Fenton-type reactions.

In mammalian cells, ferroptosis is an iron-dependent form of cell death driven by lipid peroxidation. Excess iron fuels Fenton chemistry, generating hydroxyl radicals that oxidize membrane lipids, while failure of GPx-dependent detoxification permits toxic lipid peroxide accumulation (22, 25, 45). Although bacteria lack the canonical GPx4-centered ferroptosis machinery, iron-dependent lipid peroxidation and oxidative membrane damage can still occur, suggesting the existence of a ferroptosis-like vulnerability in prokaryotes. Recent studies support this view and further indicate that such oxidative susceptibility may be therapeutically exploitable (46, 47). Notably, the marine worm-derived AMP Urechistachykinin I is among the few natural peptides reported to trigger ferroptosis-like bacterial damage, although its molecular basis remains unclear (26, 27).

Building on these considerations, and given the apparent sensitivity of MC-CATH3-mediated killing to DP, we asked whether its antibacterial activity might involve an iron-associated oxidative mechanism with features reminiscent of ferroptosis. To examine this possibility, we performed lipid peroxidation analyses using the C11-BODIPY581/591 probe and MDA quantification. MC-CATH3 markedly promoted the accumulation of intracellular lipid peroxides (Fig. 7). Although MC-CATH2 also induced lipid peroxidation, it did not show the broader set of oxidative changes observed with MC-CATH3 under our experimental conditions. HPF assays revealed that MC-CATH3 significantly increased intracellular hydroxyl radical production (Fig. 7). By contrast, MC-CATH1 produced high HPF fluorescence but did not cause lipid peroxide accumulation, suggesting that it mainly promoted peroxynitrite rather than hydroxyl radicals (Fig. 7). Further experiments showed that MC-CATH3 reduced bacterial GPx-like detoxification activity, which would be expected to impair lipid peroxide clearance and thereby favor oxidative lipid damage, a feature often associated with ferroptosis-related processes (48, 49). In contrast, MC-CATH2 enhanced GPx activity, which may help explain why it did not display the same oxidative profile despite promoting lipid oxidation (Fig. 8). Iron quantification revealed significantly elevated intracellular iron and increased Fe^2+^/Fe^3+^ ratios following MC-CATH3 treatment, consistent with the involvement of iron-associated oxidative stress (Fig. 8). Supplementation with sublethal FeCl_3_ concentrations further enhanced MC-CATH3’s killing activity, supporting a facilitatory role of iron in this process. Since iron is a cofactor for numerous essential bacterial enzymes involved in respiration, DNA synthesis, and ROS metabolism, transcriptomic analysis showed that MC-CATH3 upregulated multiple iron metabolism-related genes, including *fhuF*, *fepC*, and *fdx* (Fig. 8). Together, these observations suggest that MC-CATH3 disrupts bacterial redox and iron homeostasis, resulting in oxidative damage with features analogous to ferroptosis-like injury.

Previous research has shown that peptide hydrophobicity plays a crucial role in membrane interaction (50, 51, 52). Given MC-CATH3’s relatively low hydrophobicity, we propose that it may primarily interfere with bacterial oxidative respiration and electron transport rather than directly disrupting membrane integrity, thereby promoting excessive ROS accumulation. Experimental data support this hypothesis: MC-CATH3 treatment significantly upregulated TCA cycle-related genes, while intracellular ATP levels decreased markedly, suggesting impaired oxidative phosphorylation and reduced ETC coupling efficiency. Enzymatic assays further revealed decreased activities of complexes I and II, consistent with the drop in ATP. As these complexes contain multiple Fe-S clusters, their inactivation is consistent with oxidative damage to Fe-S centers, which could promote electron leakage and enhanced superoxide generation (Fig. 8). Correspondingly, transcriptomic data showed coordinated upregulation of genes related to energy metabolism and Fe-S cluster assembly/repair, reflecting bacterial efforts to counteract ETC dysfunction. Redox analyses revealed a decreased GSH/GSSG ratio and elevated GSSG levels, indicating depleted reducing capacity and increased oxidative stress, consistent with the observed complex I/II inactivation and Fe-S gene upregulation (Fig. 8).

**Figure 10:**
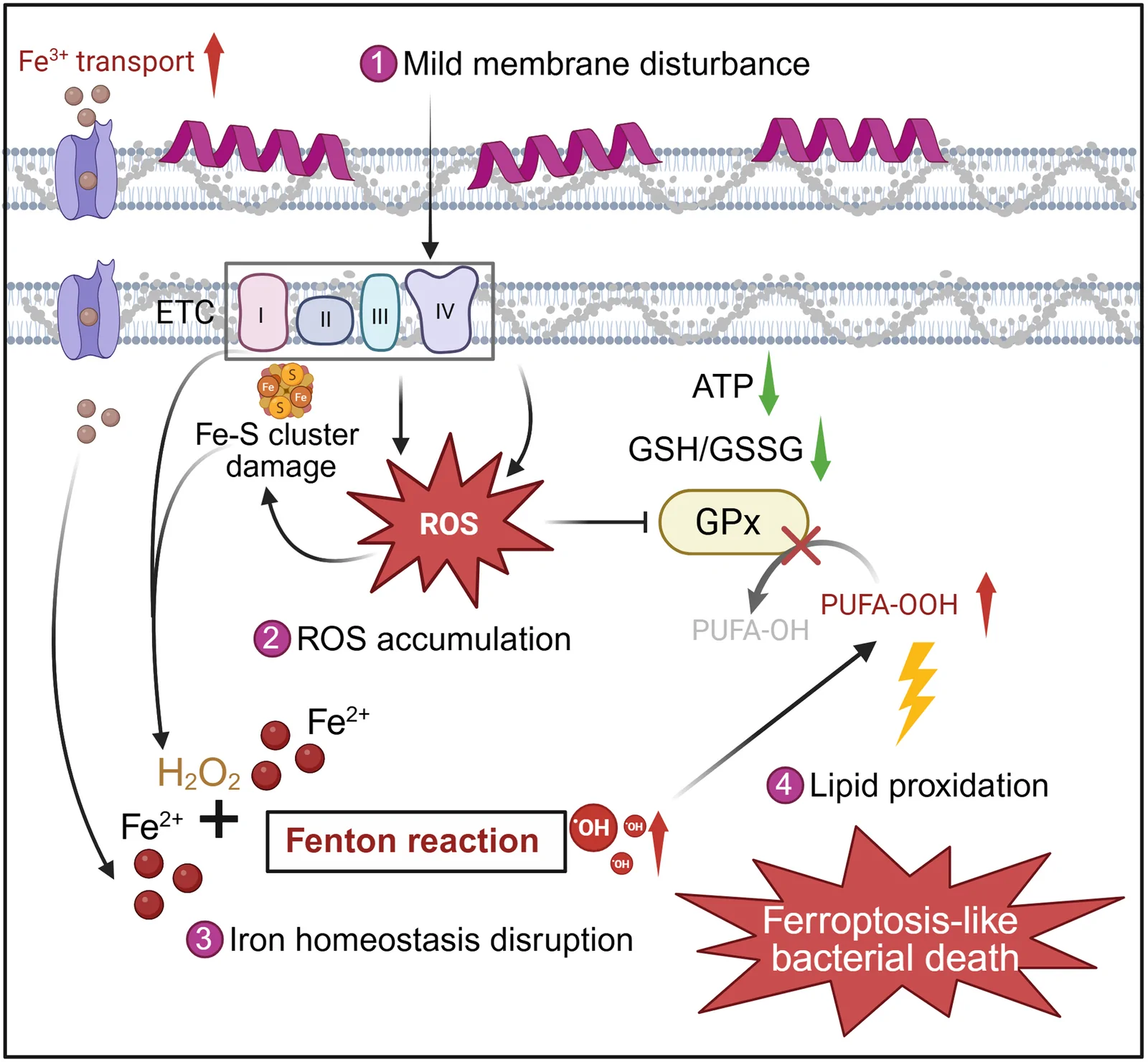
**Proposed model of MC-CATH3-triggered iron-dependent oxidative damage with ferroptosis-like features in bacteria.** MC-CATH3 interacts mildly with the bacterial membrane, causing subtle membrane disturbance that may influence ETC function and Fe-S cluster stability. These changes are proposed to promote ROS accumulation and increase the availability of catalytically active Fe^2+^. Excess Fe^2+^ may react with hydrogen peroxide (H_2_O_2_) through the Fenton reaction to generate hydroxyl radicals (•OH). At the same time, the antioxidant defense system appears to be impaired, as indicated by a decreased GSH/GSSG ratio and reduced GPx activity, which may limit ROS detoxification. Collectively, these processes could enhance lipid peroxidation in the bacterial membrane and contribute to iron-dependent oxidative damage accompanied by ferroptosis-like features.

MC-CATH3’s mode of action may therefore favor intracellular accumulation of hydrogen peroxide and superoxide anions. Subsequently, Fe^3+^ may be reduced to Fe^2+^ and participate in Fenton reactions with H_2_O_2_ to produce hydroxyl radicals. The continued buildup of hydroxyl could then contribute to oxidation of membrane PUFAs into PLOOH, while reduced GPx-like activity may further impair peroxide clearance. Taken together, these observations are consistent with bacterial death occurring through an iron-dependent oxidative process with features resembling ferroptosis. This putative ferroptosis-like process shares several features with eukaryotic ferroptosis, including iron dysregulation, ROS bursts, and substantial lipid peroxidation (Fig. 10). The multi-layered evidence presented here—including iron speciation analysis, redox imbalance profiling, GPx-like activity assessment, and transcriptomic remodeling of iron metabolism pathways—collectively supports a mechanistically connected interpretation rather than a merely correlative phenotype. These findings also argue against the likelihood that the observed lipid peroxidation being solely a secondary consequence of nonspecific oxidative injury and instead suggest that iron dysregulation is an important contributing factor. In line with this unique mechanism and its favorable cytocompatibility, MC-CATH3 effectively reduced bacterial burden, suppressed excessive inflammation, and promoted re-epithelialization and collagen-rich tissue remodeling in a murine skin wound infection model, achieving overall therapeutic efficacy comparable to vancomycin and underscoring its strong translational potential as a peptide-based anti-infective agent (Fig. 9).

At the same time, our current data do not fully exclude the possibility that some of the iron-related changes arise secondarily from broader oxidative and metabolic stress. Accordingly, we interpret iron dysregulation as an important contributor to MC-CATH3-mediated killing, while recognizing that its precise position in the causal sequence remains to be further resolved. Thus, rather than assigning iron dysregulation exclusively to an initiating event, we consider it more cautiously as a process that may both amplify oxidative damage through Fenton chemistry and emerge, at least in part, as a downstream consequence of MC-CATH3-induced stress. In particular, future studies employing bacterial mutants impaired in iron uptake or siderophore biosynthesis, such as *fepG* or *entA* knockout strains, would help to more directly assess the contribution of iron acquisition pathways to the observed phenotype. Moreover, more selective and definitive analyses of ROS species and their temporal dynamics—ideally through the use of spin traps in combination with EPR detection—as well as further investigation of peroxide detoxification systems, will be important for elucidating how MC-CATH3 disrupts redox and iron homeostasis and how these perturbations culminate in lipid oxidative damage. Such studies should help refine the mechanistic framework proposed here and more clearly define the relationship between AMP-induced bacterial killing and ferroptosis-associated features.

In summary, we identified three novel cathelicidin family AMPs from the bat *M. chinensis*. MC-CATH1 and MC-CATH2 predominantly act through membrane disruption and DNA binding, whereas MC-CATH3 displays a distinct antibacterial profile associated with iron-dependent oxidative stress, lipid peroxidation, and bacterial death with ferroptosis-related features. In addition to this mechanistic distinctiveness, MC-CATH3 exhibited favorable biocompatibility and potent *in vivo* anti-infective activity. Notably, MC-CATH3 belongs to the vertebrate cathelicidin family, distinguishing it structurally and evolutionarily from previously reported tachykinin-related peptides with similar oxidative phenotypes. The recurrence of this killing pattern across unrelated peptide classes suggests that iron-dependent oxidative bacterial killing may represent a broader antimicrobial principle. These findings expand current understanding of cathelicidin-mediated innate defense and provide a conceptual basis for developing peptide therapeutics that exploit bacterial redox and iron vulnerabilities.

## Experimental procedures

### *M. chinensis* collection and tissue preparation

Adult *M. chinensis* were captured from Tongren, Guizhou province, China. No specific permission was required for the sampling location/activity, and the present study did not involve endangered or protected species. After collection, *M. chinensis* were euthanized, and the tissues were removed quickly and stored in liquid nitrogen for later use. All animal experiments were approved by the Animal Ethics Review Committee of the Yantai Institute of Coastal Zone Research, Chinese Academy of Sciences.

### cDNA library construction and screening of cDNA encoding AMPs

The lung tissue of *M. chinensis* was ground into powder in liquid nitrogen and total RNA was extracted using Trizol reagent (Life Technologies). A cDNA library of the *M. chinensis* lung tissue was constructed using a SMART cDNA Library Construction Kit (Clotech). The experiment was conducted strictly according to the kit manual. The synthesized second-strand cDNAs was used as templates for the PCR-based cDNA screening described below (53).

According to the highly conversed cathelin domain of previously characterized mammalian cathelicidin family AMPs, a 3′ antisense primer (5′-ATTCTAGAGGCCGAGGCGGCCGACATG-3′) was designed and coupled with the 5′-sense primer (5′-AAGCAGTGGTATCAACGCAGAGTG-3′) designed based on the SMARTer V oligonucleotide primer to screen the 5' fragment of cDNAs encoding the cathelicidins of *M. chinensis*. The PCR procedure was as follow: 5 min of denaturation at 94 °C, 30 cycles of denaturation at 94 °C for 30 s, primer annealing at 58 °C for 30 s, and extension at 72 °C for 1 min. The last cycle was followed by an extension step at 72 °C for 10 min. The PCR product was purified by gel electrophoresis and cloned into pMD19-T vector (Takara, Japan) for sequencing. After the 5′ fragments of cDNA were obtained, a sense primer (5′- ATGGAGACCCAGRGSRRCAGCC-3′) was designed according to the 5′ coding region and paired with the 3′ antisense primer (5′-ATTCTAGAGGCCGAGGCGGCCGAC-3′), which was designed based on the sequence of 3′-IF SMARTer CDS primer to screen the full-length cDNAs. The PCR procedure was as follows: 5 min of denaturation at 94 °C, 30 cycles of denaturation at 94 °C for 30 s, primer annealing at 56 °C for 30 s, and extension at 72 °C for 1 min. The last cycle was followed by an extension step at 72 °C for 10 min. The PCR product was also purified by gel electrophoresis and cloned into pMD19-T vector (Takara, Japan) for sequencing.

### Bioinformatic analysis and structure modeling

The physicochemical parameters of MC-CATH1-3 were computed on the ExPASy Bioinformatics Resource Portal (http://www.expasy.org/tools/). Secondary structures of peptides were predicted by PEP-FOLD3.5 (https://mobyle.rpbs.univ-paris-diderot.fr/cgi-bin/portal.py#forms::PEP-FOLD3) (54). The top-ranked model generated by PEP-FOLD3 for each peptide was selected as the starting structure and subsequently refined using the CHARMM-GUI *PDB Reader & Manipulator* to add missing hydrogen atoms and assign protonation states at pH 7.0 (55). The final peptide structures used for peptide-bilayer interaction simulations are shown in Figure 1.

### Synthetic peptides

MC-CATH1 (GLKARAGRLPGLFGLLWDRIRNRGRRPRDVFENLSA), MC-CATH2 (GLKARVLRVNNVFRRLWDTLRNRGWRPRLFIRNLSPEEEP), and MC-CATH3 (KLNAENLGERIKNAKKKVWEKIKSFGRRIKEFFRKPSPEGEP) were synthesized by GL Biochem (Shanghai) Ltd (Shanghai, China), and analyzed by RP-HPLC and ESI-MS to ensure that the purity was higher than 95% (Figs. S8–S10). Their physicochemical parameters were shown in Table 1.

### Circular dichroism spectroscopy

Circular dichroism (CD) spectroscopy was used to evaluate the secondary structure of MC-CATH1-3 in solvent environments. The CD spectra were recorded at 298 K on a Jasco J-715 spectrophotometer (Jasco, Japan). Sample were prepared by dissolving MC-CATH1-3 to an ultimate concentration of 0.2 mg/ml in SDS/H_2_O solutions of different concentration (0, 30, 60, 90 and 120 mM). Spectra at 190 to 260 nm were measured. The instrument parameters were: 0.1 cm path-length cell, 1 nm bandwidth, 1 s response time, and a scan speed of 100 nm/min. For each sample, three consecutive scans were performed and averaged followed by subtraction of the solvent signal.

### Antimicrobial assay

To assess the antimicrobial efficacy of MC-CATH1-3, a 2-fold broth microdilution method, as detailed in our prior study (56), was employed. 13 microbial strains, comprising 6 Gram-positive bacteria, 5 Gram-negative bacteria, and two fungi, were utilized in this assay. Standard strains were maintained in our laboratory, while clinical isolates were sourced from local hospitals. In brief, microbial cultures were grown in Mueller-Hinton broth (MH broth) at 37 °C until they reached the exponential phase, then diluted to a concentration of 2 × 10^5^ CFU/ml. Serial dilutions of MC-CATH1-3 were prepared in 96-well microtiter plates (50 μl) and combined with an equal volume of microbial inoculum (50 μl). The plates were incubated in a shaking incubator at 37 °C for 18 h at 100 rpm. Microbial growth was quantified by measuring the absorbance at 600 nm at the end of the incubation period. The minimum inhibitory concentrations (MICs), defined as the lowest concentrations of MC-CATH1-3 that prevented visible microbial growth, were recorded. All MIC assays were performed in three independent experiments.

### Bacterial killing kinetic assay

The bacterial killing kinetic assay was carried out according to the method described previously with minor modifications (43). *E. coli* ATCC25922 and *S. aureus* CMCC26003 were cultured in MH broth until they reached the exponential phase, then diluted to a concentration of 1 × 106 CFU/ml with fresh MH broth. MC-CATH1-3 was added to the bacterial suspensions to achieve a final concentration of 5 × MIC. The bacteria were incubated at 37 °C for 0, 15, 30, 60, 120, and 180 min. At each time point, 50 μl samples were taken, diluted 1000-fold with fresh MH broth, and 50 μl of the diluted sample was spread on MH agar plates. The plates were incubated at 37 °C for 18 h, and the viable colonies were counted. Ampicillin and sterile PBS served as the positive and negative controls, respectively.

### Fractional inhibitory concentrations (FICs) checkerboard assay

Fractional inhibitory concentrations (FICs) were determined using checkerboard assay (57). Overnight cultures of *A. baumannii* ATCC19606 and *S. aureus* CMCC26003 were diluted to a final concentration of 1 × 10^6^ CFU/ml in MH broth at 37 °C and 200 rpm. The bacterial suspensions were then exposed to a gradient of peptide concentrations in a 96-well plate, with a total volume of 100 μl per well. The plate was incubated at 37 °C for 18 h and analyzed at a wavelength of 600 nm using a Tecan Infinite Spark microplate reader (Tecan, Switzerland). A positive control (bacterial suspension without peptides) was included in the experiment. The FIC index was calculated using the following formula:

$${\text{FIC}}_{\text{index}}={\text{MIC}}_{\text{AB}}/{\text{MIC}}_{A}+{\;\text{MIC}}_{\text{BA}}/{\text{MIC}}_{B}={\text{FIC}}_{A}+{\;\text{FIC}}_{B}$$

MIC_A_ is the MIC of compound A alone, MIC_AB_ is the MIC of compound A in combination with compound B, MIC_B_ is the MIC of compound B alone, MIC_BA_ is the MIC of compound B in combination with compound A, and FIC_A_ is the FIC of compound A and FIC_B_ is the FIC of compound B. Synergy is defined as an FIC index ≤ 0.5.

### Cytotoxic and hemolytic assay

The cytotoxic activity of MC-CATH1-3 was determined using the MTT (Sigma) method. Three mammalian cell lines (human liver hepatocellular carcinoma cell line HepG2, human embryonic kidney cell line HEK293, and mouse mononuclear macrophages cell line RAW264.7) were used in the assay, and the experiment was carried out as described in our previous work (56). Briefly, the cells were cultured in DMEM supplemented with 10% fetal bovine serum in a humidified 5% CO_2_ atmosphere at 37 °C. After digest with trypsin, the cells were diluted with serum-free DMEM to an ultimate concentration of ∼ 2.5 × 10^5^ cells/ml. The cells were seeded in 96-well plates (200 μl/well) and cultured overnight until adhesion. Subsequently, different concentrations of the peptides were added and incubated at 37 °C for 24 hours. Each well was treated with a solution containing 5 mg/ml MTT (20 μl/well) (Sigma-Aldrich, USA) and cultured for an additional 4 hours. The supernatant was removed from each well, and DMSO (200 μl per well) was added to dissolve the formazan crystals formed by the viable cells. Cell death induced by MC-CATH1-3 was expressed as the percentage of the negative control group, which was regarded as 100%.

The hemolytic assay was performed according to a previously described method (56). 1% Triton X-100 (v/v) was used to determine 100% hemolysis, and PBS was used as negative control. The hemolytic rates of MC-CATH1-3 at different concentration were recorded.

### Biofilm inhibition assay

To assess the potential effect of MC-CATH1-3 on biofilm inhibition, a modified biofilm inhibition assay was conducted (58). Briefly, 100 μl of 2 × 10^7^ CFU/ml *E coli* ATCC25922 or *S. aureus* CMCC26003 were cultured in MH broth for 24 h in a 96-well plate with varying concentrations of MC-CATH1-3. After incubation, planktonic bacteria were removed by washing the wells three times with sterile PBS, followed by fixation with methanol for 15 min. The plates were then air-dried and stained with 0.1% crystal violet (100 μl) for 20 min. Excess stain was gently rinsed off with purified water, and the stain was resolubilized in 95% ethanol. Absorbance at 600 nm was subsequently measured.

### Biofilm eradication assay

A modified biofilm eradication assay was conducted (58), the biofilms of *E. col*i ATCC25922 or *S. aureus* CMCC26003 were established in a 96-well plate by inoculating 100 μl of bacterial suspension (1 × 10^7^ CFU/ml) in MH broth. The plates were incubated at 37 °C for 24 h. Following incubation, the biofilm-containing wells were rinsed three times with PBS. MC-CATH1-3 solutions were freshly prepared in a separate 96-well plate using the same medium, and then transferred to the wells containing the biofilm suspension (100 μl each). For the negative control, 100 μl of sterile medium was added to the designated wells. The plates were subsequently incubated at 37 °C with shaking at 100 rpm for an additional 24 h. Post-incubation, the wells were emptied and washed three times with 200 μl of PBS each, followed by air-drying under aseptic conditions for 1 h. Finally, the percentage of biofilm removal was quantified by measuring the absorbance after staining with crystal violet, as previously described.

### Anti-persisters assay

The antibacterial activity of MC-CATH1-3 against persisters were assessed following a previously described method with minor modification (59). To generate persister cells from a biofilm culture, a mid-logarithmic growth-phase culture of *E. coli* ATCC25922 or *S. aureus* CMCC26003 was diluted in MH broth to 1 × 10^8^ CFU/ml. Subsequently, 100 μl of this suspension was transferred to the wells of a 96-well polystyrene flat-bottom plate. The plates were sealed and incubated at 37 °C in a humidified atmosphere for 24 h. Planktonic bacteria were removed by two washes with PBS, and biofilms were then exposed to 100 μl of MH broth containing 100 × MIC rifampicin. After another 24 h incubation at 37 °C in a humidified atmosphere, planktonic bacteria were removed by two washes with PBS, and adherent bacteria were dislodged using sonication for 5 min in 100 μl PBS. Subsequently, the bacteria were exposed to 200 μl of MH broth containing MC-CATH1-3 for 3 h, as a control group, bacteria were exposed to PBS without peptide. The bacterial suspension was serially diluted and plated onto an LB solid medium. LB solid medium was incubated in an inverted position for 18 h at 37 °C in a constant-temperature incubator. Bacterial colonies on the plates were quantified using a colony counting and measuring instrument (SupcreG10, Shineso, China). All experiments were conducted in triplicate.

### Outer membrane permeability assay

The fluorescent probe N-Phenyl-1-naphthylamine (NPN) (Invitrogen, USA) was used to evaluate the outer membrane integrity of *E. coli* ATCC25922 following treatment with MC-CATH1-3 or polymyxin B, according to a previously established protocol (60). Briefly, *E. coli* ATCC25922 was inoculated into 1 ml of MH broth and grown overnight at 37 °C with shaking at 200 rpm. Then the cultures were washed and resuspended in PBS. The optical density of the bacterial suspension at 600 nm was adjusted to 0.1 using the same buffer, and the NPN dye was added to achieve a final concentration of 5 μM. Following a 30-min incubation at 37 °C, 190 μl of the probe-labeled bacterial cells was transferred to a 96-well plate, and 10 μl of either MC-CATH1-3 or polymyxin B (at final concentrations of 1 × MIC, 2 × MIC, and 5 × MIC) was added. After a 60-min incubation, fluorescence was measured using an excitation wavelength of 350 nm and an emission wavelength of 420 nm.

### Inner membrane permeability assay

The ability of the peptides to induce bacterial cytomembrane permeabilization was determined by Propidium Iodide (PI) penetration assay as described previously with minor modifications (41). The exponential bacterial suspension of *E. coli* ATCC25922 and *S. aureus* CMCC26003 was washed and resuspended in PBS to obtain an OD600 of 0.1, followed by the addition of 10 μM of PI (Sigma-Aldrich, USA), in the presence of MC-CATH1-3. After incubation for 60 min, fluorescence was measured with the excitation wavelength at 535 nm and emission wavelength at 615 nm.

### Membrane fluidity assay

Membrane fluidity assay was conducted following an established protocol with slight modifications (43). The exponential bacterial suspension of *E. coli* ATCC25922 or *S. aureus* CMCC26003 were centrifuged and washed with PBS, then the bacterial suspensions were incubated with 10 μM Laurdan (Aladdin, China) in the dark for 30 min at 37 °C. The stained cell cultures were washed with PBS twice and concentrated. Next, different concentrations of MC-CATH1-3 were mixed with the stained bacterial cells to incubate for another 60 min at 37 °C. The Laurdan fluorescence levels were measured using a microplate reader (Tecan, Switzerland) with emission wavelengths of 440 nm and 490 nm upon excitation at 350 nm after 60 min incubation in the dark. The formula was used to calculate the final results, and the quantitation of the generalized polarization (GP) of Laurdan was used to identify the phospholipid phase.

$$\text{GP}=\left(I_{440}-I_{490}\right)/\left(I_{440}+I_{490}\right)$$

### Cytoplasmic membrane depolarization assay

DiSC_3_(5) was utilized to detect the membrane depolarization state of bacteria, following a previous protocol (61). DiSC_3_(5) was mixed in a suspension of *E. coli* ATCC25922 or *S. aureus* CMCC26003 with 3 × 10^7^ CFU/ml, and the mixture was incubated in the dark at 37 °C for 30 min. Subsequently, 190 μl of the bacterial suspension was transferred to each well of 96-well plates under black conditions, and the fluorescence intensity was measured at excitation/emission wavelengths of 622/670 nm every minute until stabilization. Finally, 10 μl of MC-CATH1-3 (final concentrations of 2 μM and 8 μM) were added to each well, and the fluorescence intensity was immediately recorded. 1% Triton X-100 with strong membrane-disrupting effects was used as a positive control, and sterile PBS was used as a negative control.

### Scanning electron microscopy (SEM) assay

The surface morphology changes in bacterial cells treated with MC-CATH1-3 were assessed using SEM (62). Specifically, overnight cultures of bacteria were washed twice and diluted to a final concentration of 1 × 10^8^ CFU/ml. These suspensions were then incubated with MC-CATH1-3 at a concentration of 5 × MIC for 60 min at 37 °C. Following incubation, the cell pellets were collected, washed twice with PBS, and fixed overnight at 4 °C in 2.5% glutaraldehyde. Subsequently, the samples were dehydrated through a graded ethanol series (30%, 50%, 70%, 90%, and 100%). The prepared samples were dried, sputter-coated with gold, and examined using an S-4800 SEM instrument (Hitachi, Japan).

### Preparation of the lipid bilayer model

Bacterial membranes typically exhibit complex lipid compositions, with phosphatidylethanolamine (PE) and phosphatidylglycerol (PG) as the predominant components (63). In this study, a lipid mixture of POPE/POPG/DPPG at a molar ratio of 80:15:5 was used to mimic inner bacteria membranes (64, 65). A symmetric bilayer containing 300 lipids per leaflet was built using the CHARMM-GUI *Membrane Builder* (66, 67, 68). A 3-nm water layer was added to each side of the bilayer to ensure full hydration, and the system was neutralized with 0.015 M KCl. The bilayer was subsequently equilibrated using the standard 6-step relaxation protocol provided by CHARMM-GUI, followed by a 100-ns production run to allow the membrane to reach thermal equilibrium (69). Simulation parameters for the production run were from the *step7_production.mdp* file generated by CHARMM-GUI (69). The coordinates of the fully equilibrated bilayer were finally extracted and used to construct the peptide–bilayer systems.

### Parameters and protocols of molecular dynamics (MD) simulations

To investigate peptide-bilayer interactions, the 3-D structure of each peptide was placed approximately 5 Å above the equilibrated POPE/POPG/DPPG bilayer surface. The system was re-solvated by adding water layers on both sides and neutralized with KCl ions. Energy minimization was performed using the steepest descent algorithm with a maximum force tolerance of 1000 kJ/mol/nm to eliminate unfavorable interactions (70). Production simulations were finally carried out in the NPT ensemble with a 2 fs time step under periodic boundary conditions. Each peptide-bilayer system was simulated in triplicate for 500 ns per replica to ensure reproducibility and quantify statistical uncertainty, resulting in a total of 4.5 μs of simulation data. Table S1 summarizes details of all simulated systems.

In addition, the temperature was maintained at 310.15 K using the Nosé-Hoover thermostat (71, 72), and the pressure (1 bar) was controlled *via* the Parrinello-Rahman barostat (73). The LINCS algorithm was used to constrain bonds involving hydrogen atoms (74). Electrostatic interactions were calculated using the Particle Mesh Ewald (PME) method with a 1.2 nm cutoff (75), and van der Waals interactions were treated using a force-switching function between 1.0 and 1.2 nm (76). All simulations employed the CHARMM36m force field (77) and TIP3P (78) water model, and were performed using GROMACS/2021.5 (70). Unless otherwise stated, all remaining parameters followed the default GROMACS settings. System visualization and snapshot rendering were carried out using VMD (79), and trajectory analyses were performed using GROMACS built-in tools, MDAnalysis (80), and MDTraj (81).

### DNA-binding assay

The DNA-binding assay was carried out in accordance with a previously described method with certain modifications (82). Genomic DNA of *E. coli* ATCC25922 was isolated using a genomic DNA extraction kit (Solarbio, China). The genomic DNA (final concentration of 10 μg/ml) was mixed with serial concentrations of MC-CATH1-3 (5, 10, 20, 40 μM) for a 30 min incubation. Sterile water was employed as the control. Subsequently, the mixture was electrophoresed through a 1% agarose gel, and DNA migration was detected *via* the fluorescence of Gel red.

The competitive binding activities of MC-CATH1-3 with ethidium bromide (EB) were evaluated using fluorescent spectrometry. The experimental method was adapted from the previous protocol with minor modifications (83). Briefly, bacterial genomic DNA from *E. coli* ATCC25922 was pre-incubated with EB (Invitrogen, USA) in sterile water at a final concentration of 1 μg/ml for 30 min at room temperature. Increasing concentrations of MC-CATH1-3 were then added to the mixture, while sterile water served as the blank control. Fluorescence detection was performed using an excitation wavelength of 535 nm and an emission wavelength range of 580 to 700 nm, with a sampling interval of 5 nm.

### Reactive oxygen species (ROS) assay

Reactive oxygen species (ROS) assay was conducted following an established protocol with slight modifications (84). The level of ROS in *E. coli* ATCC25922 and *S. aureus* CMCC26003 treated with MC-CATH1-3 and polymyxin B were quantified using 2′,7′-dichlorofluorescein diacetate (DCFH-DA) or Dihydroethidium (DHE) (Solarbio, China). Initially, bacteria were cultured overnight at 37 °C with shaking at 200 rpm, then washed and resuspended in PBS, to achieve an OD_600_ of 0.1. DCFH-DA was added to reach a final concentration of 10 μM, and the mixture was incubated at 37 °C for 30 min. Following three washes with PBS, 90 μl of probe-labeled bacterial cells was added to 96-well plate, followed by the addition of 10 μl of MC-CATH1-3 or polymyxin B. Fluorescence intensity was measured with the excitation wavelength at 488 nm and the emission wavelength at 525 nm using a Tecan Infinite Spark microplate reader (Tecan, Switzerland) after 60 min. PBS served as a negative control.

### ROS scavenge assay

The overnight cultures *E. coli* ATCC25922 and *S. aureus* CMCC26003 were collected by centrifugation at 8000 rpm for 5 min, and resuspended in MH broth to achieve a final concentration of 2 × 10^8^ CFU/ml (47). Subsequently, the bacterial suspensions were incubated with 8 μM MC-CATH1-3 for the ROS scavenger assay. For comparison, control groups were treated with 8 μM MC-CATH1-3 in the presence of either 300 mM thiourea (TU) or 80 μM 2,2-Dipyridyl (DP) for 120 min. After incubation, the cells from each group were collected by centrifugation, washed twice with PBS, and then incubated with 10 μM DCFH-DA dye in PBS at 37 °C under agitation for 30 min. The intracellular ROS levels were quantified by measuring the fluorescence intensity using a Tecan Infinite Spark microplate reader (Tecan, Switzerland) at excitation/emission wavelengths of 488/525 nm.

### RNA-seq and transcriptomic analyses

*E. coli* ATCC25922 was cultured in MH broth at 37 °C and 200 rpm using a THZ-100 constant temperature culture shaker until reaching the logarithmic growth phase. The culture was subsequently diluted 1:10 in fresh MH broth supplemented with MC-CATH1-3 at a concentration equivalent to 1 × MIC. After a 2-h incubation, the bacteria were washed three times with PBS and collected by centrifugation at 7000 rpm for 10 min at 4 °C. A prokaryotic RNA library was then constructed and sequenced by Sango Biotechnology Co., Ltd, according to a previously published protocol (85). The Ribo-off rRNA Depletion Kit (Vazyme, China) was employed to remove ribosomal RNA (rRNA) from the total RNA. The VAHTS Stranded mRNA-seq V2 Library Prep Kit for Illumina (Vazyme, China) was then utilized to construct a sequencing library from the rRNA-depleted RNA. The purified complementary DNA (cDNA) library was sequenced on the Illumina HiSeq platform, producing 150 bp paired-end reads. The short reads were mapped to reference sequences obtained from the NCBI database. Transcript per million (TPM) was used to quantify gene expression levels. Differentially expressed genes (DEGs) were identified using DESeq1.26.0 software, with criteria of q < 0.05 and an absolute log fold change ≥ 1. Gene Ontology (GO) and Kyoto Encyclopedia of Genes and Genomes (KEGG) enrichment analyses were performed to explore the pathways and biological phenomena associated with the DEGs. The raw RNA-seq data generated in this study have been deposited in the NCBI Sequence Read Archive under BioProject accession number PRJNA1494787.

### Lipid peroxidation detection

Lipid peroxidation detection was conducted following an established protocol with slight modifications (86, 87). The overnight cultures *E. coli* ATCC25922 and *S. aureus* CMCC26003 were collected and resuspended in MH broth to achieve a final concentration of 1 × 10^7^ CFU/ml and co-cultured with 5 × MIC of MC-CATH1-3 for 60 min. Following this, the bacteria were washed and resuspended in MH broth. Suspensions of *E. coli* ATCC25922 and *S. aureus* CMCC26003 were incubated in darkness at 37 °C for 30 min with 10 μM C11-BODIPY (581/591) (Cayman Chemical, Germany). Fluorescence intensity was measured using a microplate reader, specifically the oxidized C11-BODIPY (581/591) fluorescence intensity with excitation at 488 nm and emission at 510 nm. A negative control was performed using MH broth.

Malondialdehyde (MDA) levels, as markers of lipid peroxidation and oxidative stress leading to cell death, were quantified using the MDA assay kit (Solarbio, China) (88). The cells treated with MC-CATH1-3 were incubated for 60 min at 37 °C. Following this, the bacterial cells were washed, and the MDA concentration was determined according to the manufacturer's instructions.

### RNA isolation and qPCR

Total RNA was extracted from peptide-treated *E. coli* cells using the Total RNA Kit I (OMEGA) according to the manufacturer’s instructions. One microgram of RNA was reverse transcribed into cDNA using the Goldenstar RT cDNA Synthesis Mix (TsingKe Biotech). Quantitative real-time PCR (qPCR) was performed with SYBR Green Master Mix (MCE) on a real-time PCR system. Primer sequences used for amplification are listed in Table S2. Gene expression levels were normalized to the internal reference gene and calculated using the 2^−ΔΔCt^ method (89).

### Detection of hydroxyl radical

Hydroxyl radicals are toxic to cells in various aspects. The levels of Hydroxyl radicals were measured using 3′-p-Hydroxyphenyl fluorescein (HPF) (Cayman Chemical). This method was adapted from a previously described protocol with minor modifications (90). Briefly, *E. coli* ATCC25922 and *S. aureus* CMCC26003 were incubated with MC-CATH1-3 for 60 min. The samples were then centrifuged at 7000 rpm for 5 min, and the supernatant was discarded. PBS containing 10 μM HPF was added, and the mixture was incubated for 30 min in the dark at 37 °C. Fluorescence intensity was subsequently analyzed. The excitation and emission wavelengths for HPF were 490 nm and 515 nm, respectively.

### Phospholipid inhibition assay

The impact of exogenous lipopolysaccharide (LPS) and various lipids on the activity of MC-CATH1-3 was evaluated using the 2-fold microdilution assay, following an established protocol with minor modifications (62). Briefly, LPS (0–128 μg/ml), phosphatidylglycerol (PG, 0–128 μg/ml), phosphatidylethanolamine (PE, 0–128 μg/ml), cardiolipin (CL, 0–128 μg/ml), phosphatidylcholine (PC, 0–128 μg/ml) and MC-CATH1-3 were co-incubated with bacterial suspensions in 96-well plates. After 16 to 18 h of incubation, the MICs of MC-CATH1-3 in the presence of LPS and different lipids were determined.

### GSH/GSSG ratio afssay

After treatment with MC-CATH1-3, *E. coli* ATCC25922 and *S. aureus* CMCC26003 were incubated for 60 min at 37 °C. Bacterial pellets were harvested, washed twice with ice-cold PBS, and lysed by ultrasonication on ice. The clarified supernatant was collected for glutathione analysis. The intracellular levels of reduced glutathione (GSH) and oxidized glutathione (GSSG) were determined using the GSH/GSSG Assay Kit (Beyotime, China) following the manufacturer’s instructions. Briefly, GSH reacts with DTNB [5,5′-dithiobis-(2-nitrobenzoic acid)] to generate a yellow product with an absorbance peak at OD_412_, while GSSG was quantified after masking free GSH with a scavenger and then enzymatically reducing GSSG back to GSH. The GSH/GSSG ratio was calculated as an indicator of cellular redox status. All measurements were performed in triplicate, and results were normalized to total protein content determined by the BCA assay.

### Glutathione peroxidase (GPx) activity assay

GPx activity was assessed based on a previously published method with minor modifications (91). Specifically, GPx catalyzes the oxidation of GSH by H_2_O_2_ to produce GSSG, which can generate compounds with DTNB with a characteristic absorption peak at 412 nm, and the decrease of absorbance at 412 nm can reflect the activity of GPx. The GPx assay kit (Solarbio, China) was utilized to quantify GPx activity. *E. col*i ATCC25922 and *S. aureus* CMCC26003 were treated with MC-CATH1-3 and incubated for 60 min at 37 °C. Following bacterial cell washing, GPx activity was evaluated according to the manufacturer's instructions provided with the GPx assay kit.

### Respiratory chain complex I and II activity assay

After treatment with MC-CATH1-3, *E. coli* ATCC25922 and *S. aureus* CMCC26003 were incubated for 60 min at 37 °C. Bacterial pellets were collected, washed twice with ice-cold PBS, and lysed by ultrasonication on ice. The clarified supernatant was used for enzyme activity measurements. The activities of respiratory chain Complex I (NADH dehydrogenase) and Complex II (succinate dehydrogenase) were determined using the Mitochondrial Respiratory Chain Complex I and II Activity Assay Kits (Solarbio) following the manufacturer’s instructions. Briefly, Complex I activity was assessed by monitoring the NADH-dependent reduction of a colorimetric electron acceptor, while Complex II activity was measured based on the succinate-dependent reduction of an artificial electron carrier. Absorbance changes were recorded at OD_340_ (Complex I) and OD_600_ (Complex II) using a microplate reader, and enzyme activities were calculated according to kit guidelines. All measurements were performed in triplicate and normalized to total protein content quantified by the BCA assay.

### Intracellular iron level detection

After treatment with MC-CATH1-3, *E. coli* ATCC25922 and *S. aureus* CMCC26003 were incubated for 60 min at 37 °C. Bacterial pellets were collected and thoroughly washed with PBS to remove extracellular iron. The cells were then lysed by ultrasonication on ice, and the supernatant was collected for analysis. Intracellular iron levels were determined using the Cellular Iron Content Assay Kit (Solarbio, China) according to the manufacturer’s instructions. Briefly, hydroxylamine hydrochloride was added to reduce Fe^3+^ to Fe^2+^ under weakly acidic conditions, allowing Fe^2+^ to form a stable orange-red complex with o-phenanthroline. The reaction mixture was incubated for 10 min at room temperature, and absorbance was measured at 510 nm using a microplate reader. For differential detection, Fe^2+^ content was measured directly using ferrozine at 562 nm, while total iron was determined after reduction with hydroxylamine hydrochloride. For ICP-MS analysis, equal amounts of bacterial pellets were thoroughly washed with metal-free buffer, transferred into acid-washed tubes, and digested with ultrapure nitric acid at 80 to 100 °C until the solutions became clear. After cooling, the digests were brought to a defined volume with ultrapure water, and iron levels in the samples were determined by inductively coupled plasma mass spectrometry (ICP-MS). All experiments were performed in triplicate, and data were normalized to bacterial counts or total protein content.

### Mouse skin wound infection model

Female BALB/c mice (6–8 weeks, 18–22 g) were purchased from Shandong Pengyue Experimental Animal Technology Co., Ltd and maintained in a SPF animal facility with free access to food and water. All procedures were approved by the Animal Ethics Review Committee of the Yantai Institute of Coastal Zone Research, Chinese Academy of Sciences. Under isoflurane anesthesia, dorsal hair was shaved and disinfected with 75% ethanol, and a full-thickness circular wound was created on the back using a sterile biopsy punch. The wound surface was inoculated with 50 μl of a *S*. *aureus* CMCC26003 suspension (1 × 10^8^ CFU/ml in PBS). From the following day, mice were randomly assigned to treatment groups (*n* = 8 per group) and received 50 μl of PBS, vancomycin (5 mg/kg), or MC-CATH1, MC-CATH2, or MC-CATH3 (5 mg/kg), applied dropwise onto the wound once daily for three consecutive days. Wound healing was monitored by digital photography, and wound areas were quantified using ImageJ. On days 3 and 6, the wound surface was gently rinsed with PBS, and the lavage fluid was serially diluted and plated on agar to determine bacterial counts (CFU/ml). On day 6, blood samples were collected and serum levels of IL-6 and TNF-α were measured using commercial ELISA kits (Invitrogen). On day 12, mice were euthanized and dorsal skin tissues surrounding the wound were harvested, fixed, embedded in paraffin, sectioned, and stained with H&E and Masson’s staining for histological analysis.

## Quantification and statistical analysis

All data were analyzed using GraphPad Prism 8.0 (GraphPad Software Inc., San Diego, CA, USA) and presented as the mean ± standard deviation (SD). An unpaired *t* test was employed to assess differences between groups. Statistical significance was denoted as follows: non-significant (ns) for *p* > 0.05, ∗ for *p* < 0.05, ∗∗ for *p* < 0.01, ∗∗∗ for *p* < 0.001, and ∗∗∗∗ for *p* < 0.0001, with increasing levels of significance.

## Data availability

All data are included in the article and supplementary information files. The raw RNA-seq data generated in this study have been deposited in the NCBI Sequence Read Archive under BioProject accession number PRJNA1494787.

## Supporting information

This article contains supporting information.

## Conflict of interest

The authors declare that they have no conflicts of interest with the contents of this article.
