# Supplementary material for: Bat-derived antimicrobial peptide MC-CATH3 Confers protection against bacterial infections via a potential ferroptosis-like death process
Source: J Biol Chem. 2026 Aug 17;302(10):113456. doi: 10.1016/j.jbc.2026.113456 (PMC13579941; doi:10.1016/j.jbc.2026.113456)
Supplement: Supplementary file 1 [file mmc1.doc]

**Supplementary Information**

**Bat-Derived Antimicrobial Peptide MC-CATH3 Confers Protection Against Bacterial Infections via a Potential Ferroptosis-Like Death Process**

Xiangjin Kong^1,2,‡^, Zhouye Xu^3,‡^, Jinhui Li^4^, Zhizhen Liu^1,5^, Yiyun Zhu^3^, Zifan Ye^1,3^, Shuangyu Li^1,2^, Jingjing Zhang^1,2^, Lei Fu^4,*^, Xudong Jiao^1,2,*^, Yipeng Wang^1,2,*^

^1^Yantai Institute of Coastal Zone Research, Chinese Academy of Sciences; Yantai, Shandong 264003, China.

^2^University of Chinese Academy of Sciences; Beijing 100049, China.

^3^Department of Biopharmaceutical Sciences, College of Pharmaceutical Sciences, Soochow University; Suzhou, Jiangsu 215123, China.

^4^Institute of Medical Artificial Intelligence, South China Hospital, Medical School, Shenzhen University; Shenzhen,Guangdong 518116, China.

^5^School of Biological Science and Technology, University of Jinan; Jinan, Shandong 250022, China.

^‡^These authors contribute equally.

^*^Correspondence and material requests should be addressed to L.F. (email: leifu@szu.edu.cn), X.D.J. (email: [xdjiao@yic.ac.cn](mailto:xjkong@yic.ac.cn)), and Y.P.W. (email: [ypwang@yic.ac.cn](mailto:ypwang@yic.ac.cn)).

**Supplementary Figure 1. cDNA sequences encoding the MC-CATHs and predicted prepropeptide sequences.** Putative mature peptides of MC-CATHs are boxed. Stop codon is indicted by an asterisk (*).

**Supplementary Figure 2.** **Synergistic antimicrobial activity of MC-CATHs. A**-**C**, Checkerboard assays evaluating pairwise combinations of MC-CATH peptides against *A. baumannii* ATCC19606. **D**-**F**, Checkerboard assays evaluating pairwise combinations of MC-CATH peptides against *S. aureus* CMCC26003. In the heatmaps, darker red or blue regions represent higher bacterial densities and lower inhibition rates of the peptide combinations, whereas lighter/more transparent regions represent lower bacterial densities and stronger inhibition; white regions indicate no detectable bacterial growth. Synergy was evaluated by calculating the fractional inhibitory concentration index (FICI), with FICI ≤ 0.5 indicating synergism.

**Supplementary Figure 3. Cytotoxicity, hemolysis, and stability of MC-CATHs**. **A**-**C**, Cytotoxicity of MC-CATHs toward HEK293 (**A**), RAW264.7 (**B**), and HepG2 (**C**) cells. Cells were incubated with gradient peptide concentrations for 24 h, followed by MTT assays to assess viability. **D**, Hemolytic activity of MC-CATHs. Mouse red blood cells were incubated with gradient peptide concentrations for 30 min, with 1% Triton X-100 serving as a positive control. All experiments were performed in triplicate, and data are presented as mean ± SD. Statistical significance was determined by unpaired t-test (ns: not significant; **p* < 0.05; ***p* < 0.01; ****p* < 0.001; *****p* < 0.0001).

**Supplementary Figure 4.** Time series of root-mean-square deviation (RMSD) for MC-CATH1 (**A**), MC-CATH2 (**B**), and MC-CATH3 (**C**) in their corresponding peptide-bilayer systems.

**Supplementary Figure 5.** Time series of nonbonded interaction energies of MC-CATH1 (**A**), MC-CATH2 (**B**), and MC-CATH3 (**C**) with water and bilayer.

**Supplementary Figure 6.** **Oxidative stress contributes to the antibacterial activity of MC-CATHs. A**, Intracellular ROS assays (DCFH-DA fluorescence) in *S. aureus* exposed to MC-CATH1-3 with TU (300 mM) or DP (80 μM). MC-CATHs were applied at 8 μM, and the probe concentration was 10 μM. **B**, Representative colony images showing the effects of TU or DP on *S. aureus* survival following MC-CATH treatment. MC-CATHs were applied at a final concentration of 8 μM; DP and TU were used at final concentrations of 80 μM and 300 mM, respectively. **C**, Lipid peroxidation levels in *S. aureus* treated with MC-CATH peptides were determined using the lipid peroxidation probe C11-BODIPY581/591. MC-CATHs were applied at 0.25×-4× MIC, and the probe concentration was 10 μM. **D**, Malondialdehyde (MDA) levels in *S. aureus* following peptide treatment. MC-CATHs were applied at 5× MIC. **E**, Reactive oxygen species (ROS) generation in *S. aureus* as a function of MC-CATHs concentration was evaluated using HPF fluorescence intensity. MC-CATHs were applied at 0.25×-4× MIC, and the probe concentration was 10 μM. All experiments were performed in triplicate, and data are presented as mean ± SD. Statistical significance was assessed by unpaired t-test (ns: not significant; **p* < 0.05; ***p* < 0.01; ****p* < 0.001; *****p* < 0.0001).

**Supplementary Figure 7.** **Cross-species validation of the iron-dependent oxidative mechanism in *S. aureus* CMCC26003.** **A**, Intracellular ATP concentration measured in untreated control cells and cells exposed to MC-CATHs. MC-CATHs were applied at 5× MIC. **B**, Mitochondrial respiratory chain activity determined for complex I and complex II following peptide exposure. MC-CATHs were applied at 5× MIC. **C**, Cellular redox balance shown as the ratio of reduced glutathione (GSH, red) to oxidized glutathione (GSSG, blue), expressed as percentage, across the same experimental conditions. MC-CATHs were applied at 5× MIC. **D**, Glutathione peroxidase (GSH-Px/GPx) enzymatic activity quantified in control and peptide-treated groups. MC-CATHs were applied at 5× MIC. **E**, Total intracellular iron content in each treatment group. MC-CATHs were applied at 5× MIC. **F**, Intracellular iron accumulation quantified by inductively coupled plasma mass spectrometry (ICP-MS) following MC-CATH treatment. MC-CATHs were applied at 5× MIC. **G**, Distribution of ferrous (Fe²⁺, red) and ferric (Fe³⁺, blue) iron species shown as percentage (%), indicating changes in intracellular iron redox state after peptide treatment. MC-CATHs were applied at 5× MIC. **H**, Effect of exogenous addition of FeCl_3_ on the antibacterial activity of MC-CATHs. MC-CATHs were applied at 2× MIC, and FeCl₃ was added at a final concentration of 32 μM. **I**, Representative dilution plating assay showing the survival of S. aureus after MC-CATH treatment in the presence or absence of exogenous FeCl₃. **J**, Quantification of viable bacteria from the dilution plating assay under the indicated treatment conditions. For **H-J**, MC-CATHs were applied at 2× MIC, and FeCl₃ was added at a final concentration of 32 μM. All experiments were performed in triplicate, and data are presented as mean ± SD. Statistical significance was assessed by unpaired t-test (ns: not significant; **p* < 0.05; ***p* < 0.01; ****p* < 0.001; *****p* < 0.0001).

**Supplementary Figure 8.** The purity and identity of MC-CATH1 were confirmed by HPLC (**A**) and ESI-MS (**B**).

**Supplementary Figure 9.** The purity and identity of MC-CATH2 were confirmed by HPLC (**A**) and ESI-MS (**B**).

**Supplementary Figure 10.** The purity and identity of MC-CATH3 were confirmed by HPLC (**A**) and ESI-MS (**B**).

**Supplementary Table 1**. Summary of simulated peptide-bilayer systems.

| Peptides | Lipids/  Leaflet | Replica # | Water # | Total Atom # | Simulation Box Sizes (nm) | Simulation Length |
| --- | --- | --- | --- | --- | --- | --- |
| MC-CATH1 | 300 | 1 | 29826 | 165615 | 13.2 × 13.2 × 9.2 | 500 ns |
|  |  | 2 | 29738 | 165353 | 13.2 × 13.2 × 9.3 | 500 ns |
|  |  | 3 | 29381 | 164282 | 13.2 × 13.2 × 9.2 | 500 ns |
| MC-CATH2 | 300 | 1 | 29769 | 165557 | 13.2 × 13.2 × 9.3 | 500 ns |
|  |  | 2 | 29686 | 165310 | 13.1 × 13.1 × 9.4 | 500 ns |
|  |  | 3 | 29336 | 164260 | 13.2 × 13.2 × 9.2 | 500 ns |
| MC-CATH3 | 300 | 1 | 29724 | 165436 | 13.3 × 13.3 × 9.2 | 500 ns |
|  |  | 2 | 29712 | 165402 | 13.1 × 13.1 × 9.4 | 500 ns |
|  |  | 3 | 29335 | 164271 | 13.2 × 13.2 × 9.1 | 500 ns |

**Supplementary Table 2**. Oligonucleotides primers used in the current study.

| Primers | Sequence (5’-3’) |
| --- | --- |
| Q-*phoP*-F | GCGCGTACTGGTTGTTGAAG |
| Q-*phoP*-R | AATGGTATGGCTTTCCCGCA |
| Q-*pmrD*-F | CTTATGCTCTGCGATGCTGG |
| Q-*pmrD*-R | TCCCTGCCACTTTACACTGC |
| Q-*arnT*-F | TGCATCTCTGGCAATGCTGA |
| Q-*arnT*-R | CACCGCTGACAAATCTCCCT |
| Q-*pgpC*-F | GAGCGTCGTGTGGTGTTTTT |
| Q-*pgpC*-R | AAAGCAGCGGATTGTCCTGA |
| Q-*sucA*-F | TCCTGCAGCTCATTAACGCA |
| Q-*sucA*-R | ATGTTTACCGGCGAACTCGT |
| Q-*Fur*-F | ACCGCCCTAAAGAAAGCTGG |
| Q-*Fur*-R | TATTTGCCTTCGTGCGCATG |
| Q-*sufA*-F | GCAAGGCTTAACGCTGACAC |
| Q-*sufA*-R | ATACCCCAAAGCTTTCGCCA |
| Q-*sodC*-F | TAGTCTGGCTATTCTGGCGC |
| Q-*sodC*-R | TACACCACAGGCATAGCGTT |
| Q-*hyaA*-F | CCCAGGCGGAAGAAGTCTTT |
| Q-*hyaA*-R | GTGTGGAGGAACAGGCGTTA |
| Q-*fepC*-F | GCGAACAGTTAACCCTGGGA |
| Q-*fepC*-R | GCCTTTTTCGCGGTTCAGTT |
| Q-*fdx*-F | GCCTCATCAGGATCTCTGCC |
| Q-*fdx*-R | CACGCGCATGGTTGATAGTG |
| Q-*sufS*-F | CAGAAACCGAGCCAGGTGAT |
| Q-*sufS*-R | GCCATCCACCAGCACTTTTG |
| Q-*16S*-F | GGAACTGAGACACGGTCCAG |
| Q-*16S*-R | TTTAACCTTGCGGCCGTACT |
